# A structural heart-brain axis mediates the association between cardiovascular risk and cognitive function

**DOI:** 10.1162/imag_a_00063

**Published:** 2024-01-25

**Authors:** Akshay Jaggi, Eleanor L.S. Conole, Zahra Raisi-Estabragh, Polyxeni Gkontra, Celeste McCracken, Liliana Szabo, Stefan Neubauer, Steffen E. Petersen, Simon R. Cox, Karim Lekadir

**Affiliations:** Facultat de Matemàtiques i Informàtica, Universitat de Barcelona, Barcelona, España; Lothian Birth Cohorts, Department of Psychology, The University of Edinburgh, Edinburgh, United Kingdom; William Harvey Research Institute, NIHR Barts Biomedical Research Centre, Queen Mary University of London, Charterhouse Square, London, United Kingdom; Barts Heart Centre, St Bartholomew’s Hospital, Barts Health NHS Trust, West Smithfield, London, United Kingdom; Division of Cardiovascular Medicine, Radcliffe Department of Medicine, University of Oxford, National Institute for Health and Care Research Oxford Biomedical Research Centre, Oxford University Hospitals NHS Foundation Trust, Oxford, United Kingdom; Health Data Research UK, London, United Kingdom; Alan Turing Institute, London, United Kingdom; Institució Catalana de Recerca i Estudis Avançats (ICREA), Passeig Lluís Companys 23, Barcelona, Spain

**Keywords:** heart-brain axis, cognitive decline, cardiovascular disease, ageing, structural MRI, imaging-derived phenotypes

## Abstract

Elevated vascular disease risk associates with poorer cognitive function, but the mechanism for this link is poorly understood. A leading theory, the structural-functional model argues that vascular risk may drive adverse cardiac remodelling, which, in turn, leads to chronic cerebral hypoperfusion and subsequent brain structural damage. This model predicts that variation in heart and brain structure should associate with both greater vascular risk and lower cognitive function. This study tests that prediction in a large sample of the UK Biobank (N = 11,962). We assemble and summarise vascular risk factors, cardiac magnetic resonance radiomics, brain structural and diffusion MRI indices, and cognitive assessment. We also extract “heart-brain axes” capturing the covariation in heart and brain structure. Many heart and brain measures partially explain the vascular risk—cognitive function association, like left ventricular end-diastolic volume and grey matter volume. Notably, a heart-brain axis, capturing correlation between lower myocardial intensity, lower grey matter volume, and poorer thalamic white matter integrity, completely mediates the association, supporting the structural-functional model. Our findings also complicate this theory by finding that brain structural variation cannot completely explain the heart structure—cognitive function association. Our results broadly offer evidence for the structural functional hypothesis, identify imaging biomarkers for this association by considering covariation in heart and brain structure, and generate novel hypotheses about how cardiovascular risk may link to cognitive function.

## 1 Introduction

With ageing populations throughout the world, cognitive decline now affects an increasingly large portion of society and contributes to significant financial burden and death (Gorelick et al., 2017; Wimo et al., 2013). Of the drivers of age-related cognitive decline, neurovascular health has gained attention due to its widespread impact and relative ease of intervention (Gorelick et al., 2011; Iadecola et al., 2016; Qiu & Fratiglioni, 2015; Sweeney et al., 2018).

Substantial work has shown diverse associations between vascular disease risk factors (VRFs, such as diabetes, high body mass index (BMI), and hypertension) and cognitive function (CF). Greater vascular risk in middle and old age associates with both poorer cognitive function and accelerated cognitive decline (Jefferson et al., 2010; Knopman et al., 2001; Lyall et al., 2017; Samieri et al., 2018; Yaffe et al., 2014), and controlling vascular risk factors can lead to a decrease in onset of mild cognitive impairment (SPRINT MIND Investigators for the SPRINT Research Group et al., 2019).

Better understanding of the mechanism of this heart-brain axis will facilitate biomarker development and treatment discovery for neurovascular health. Several mechanistic theories exist but lack evidence (Jensen et al., 2023; M. Wang et al., 2016; Zenger et al., 2023). One popular model, the structural-functional model, argues that VRFs might drive pathologic cardiac and cerebrovascular remodelling, which could then result in chronic cerebral hypoperfusion, brain structural damage, and poorer CF (de la Torre, 2012a, 2012b; Pasha et al., 2017; Qiu & Fratiglioni, 2015; van Buchem et al., 2014). Direct evidence for this theory has remained unclear but could be found by simultaneously measuring vascular risk factors, cognitive function, and heart and brain structure.

Cardiac and brain imaging derived phenotypes (IDPs) have become popular methods for measuring heart and brain structure due to their minimally invasive nature and widespread use. Both are strong candidate biomarkers of the modest but well-replicated association between elevated vascular risk and lower cognitive function in middle and older age (Ferguson et al., 2020; Lyall et al., 2017). However, to date, most of our knowledge about associations between (1) VRFs, (2) cardiac structure, (3) brain structure, and (4) cognitive measures come from separate reports, which only simultaneously consider two phenotypes of interest (Cox, Lyall, et al., 2019; Lyall et al., 2017; McCracken et al., 2021; Raisi-Estabragh, Jaggi, et al., 2021; Raisi-Estabragh, M’Charrak, et al., 2021). Several recent works have indicated the value in extending analyses across three of the four phenotype categories above (Bai et al., 2020; McCracken et al., 2022; Newby et al., 2021; Shen et al., 2020); for example, lower grey matter volume can explain part of the association between hypertension, greater BMI, and lower performance on some UK Biobank cognitive exams (Ferguson et al., 2020). Most recently, a large scale study has revealed that cardiac imaging features, brain imaging features, and neuropsychiatric disease all share a common genetic influence, motivating further work in exploring how these systems may interact physiologically (Zhao et al., 2023). However, none of these studies have specifically studied how inter-relations between cardiac and brain structural variation could explain the vascular risk - cognitive function association.

We hypothesise that, for the structural-functional model to adequately explain the VRF-CF association, separate heart and brain structures should associate with both greater vascular risk and lower cognitive function. In other words, heart and brain structural variation should mediate the VRF-CF association (Bai et al., 2020; McCormick et al., 2022). Additionally, heart mediators should associate with brain mediators. Finally, for all steps of the structural-functional model to be supported by the data, heart structural variation should mediate the VRF-brain structure association, and brain structural variation should mediate the heart structure–CF association. The extent to which these associations all align in a cohort of subjects modelled together is understudied (Ferguson et al., 2020; Gorelick & Sorond, 2018; Newby et al., 2021). Furthermore, the relative strength of the association between cardiac and brain structural features and the disease endpoints (vascular risk and cognitive decline) is unknown. Along with validating the structural functional hypothesis, this comparative approach could identify novel biomarkers associated specifically with the VRF-CF association (rather than each dataset alone) and guide future decision-making comparing and prioritising organ-specific interventions in vascular and cognitive health (Banus et al., 2021; Gorelick & Sorond, 2018; Gorelick et al., 2011, 2017).

To test the structural-functional hypothesis, in this work, we measure the extent that variation in heart and brain structure explains the association between vascular risk and cognitive function in the UK Biobank. We gather vascular risk factors, cognitive exam performance, cardiac magnetic resonance imaging (CMR) radiomics features, and brain MRI IDPs for 11,962 UK Biobank participants. We perform dimensionality reduction on all datasets separately. We discover novel measures of the heart-brain axis by capturing correlated variance in heart and brain imaging. We compute single and multiple mediation models asking how well imaging latent variables explain the VRF-CF association. We then measure how well imaging latent variables explain associations between individual VRFs and cognitive exams. We finally explore how well individual heart and brain structural measures mediate the VRF-CF association. Along with myriad smaller mediating effects, we find that myocardial intensity, grey matter volume, and thalamic white matter tract integrity all associate with each other, and a joint factor capturing their variability most strongly associates with both elevated vascular risk and poorer cognitive function.

## 2 Methods

### 2.1 Acquisition and processing

#### 2.1.1 Assessment

This work utilises clinical and imaging data from the United Kingdom (UK) Biobank via access application 2964 (Ukbb-Prot-, n.d.). The UK Biobank is a large-scale longitudinal dataset derived from 500,000 volunteers recruited between 2006 and 2010 from across the UK. At visits, participants completed both a touchscreen questionnaire and medical history interview with a nurse. The project recorded information regarding participants’ health, lifestyle, and family history and collected physical measurements, biological samples, and genome. Moreover, since 2015, over 50,000 participants have received CMR and brain MR imaging at follow-up imaging visits.

#### 2.1.2 Vascular risk factors

We analysed hypercholesterolemia, diabetes, hypertension, smoking pack years, blood pressure, and anthropomorphic measures (BMI and waist-to-hip ratio, WHR) (Cox, Lyall, et al., 2019; de la Torre, 2012a, 2012b; Haley et al., 2018; Pasha et al., 2017; Sweeney et al., 2018). All vascular risk factors were collected at the baseline visit and prepared as reported previously (Cox, Lyall, et al., 2019; Raisi-Estabragh, Jaggi, et al., 2021). We summarise the process here. Diagnosis of diabetes, hypertension, and hypercholesterolemia was established via a combination of self-report, biochemistry, and linked hospital episode statistics (HES) data (Supplementary Methods) (Raisi-Estabragh, Jaggi, et al., 2021). Participants provided information on cigarette smoking in the touchscreen questionnaire, and smoking pack years were computed from this data (Cox, Lyall, et al., 2019). Blood pressure was collected twice, moments apart, using an Omron 705IT monitor. Mean systolic and diastolic blood pressure were computed. Anthropometric measures were taken after participants had removed bulky clothing and shoes. Waist and hip measurements were conducted to provide WHR. BMI was computed by dividing weight by squared height.

#### 2.1.3 Cognitive exams

Cognitive testing was performed at both the UK Biobank baseline and imaging sessions; we examined four tests from the imaging visit cognitive assessment. The complete battery and assessment of its repeatability and reliability have been detailed previously (Fawns-Ritchie & Deary, 2020; Lyall et al., 2016, 2017). We used the four tests commonly used in analysis and dimensionality reduction of the baseline cognitive assessment: the fluid intelligence task (verbal numerical reasoning, VNR), the visual memory task (vismem), the reaction time task (RT), and the prospective memory task (prosmem) (Lyall et al., 2016). As previously reported (Lyall et al., 2016), the reaction time scores were positively skewed, so we applied a natural log transformation (LN). Additionally, the visual memory scores were zero-inflated and positively skewed, so we applied an LN+1 transformation.

#### 2.1.4 Cardiac imaging

Cardiac imaging acquisition and preparation are discussed in Supplementary Methods. Using the CMR images and their corresponding segmentations, we performed radiomics phenotyping based on the open-source python-based pyradiomics library (van Griethuysen et al., 2017). Radiomics extracts features quantifying myocardial and ventricular structure (shape radiomics), myocardial imaging intensity (first-order radiomics), and myocardial visual textures (texture radiomics) (Raisi-Estabragh et al., 2020). In total, 212 features per region were extracted at end-diastole and end-systole. Right and left ventricular cavity first-order and texture features were excluded from analysis because they do not encompass clinically relevant information. We also incorporate alternative traditional and advanced CMR indices into the matching analysis and final mediation by individual features, computed as previously reported (detailed in Supplementary Methods) (Bai et al., 2020; McCracken et al., 2021; Raisi-Estabragh, McCracken, et al., 2021; Raisi-Estabragh, M’Charrak, et al., 2021; Zhao et al., 2023).

#### 2.1.5 Brain imaging

Brain imaging acquisition and preparation is discussed in Supplementary Methods. The global tissue volumes and white matter tract-averaged water molecular diffusion indices were processed by the UK Biobank team and made available to approved researchers as imaging-derived phenotypes (IDPs); the full details of the image processing and QC pipeline are available in an open access article (Alfaro-Almagro et al., 2018). The IDPs in this study included total brain volume, grey matter volume, subcortical volumes, and tract-averaged white matter microstructural measures. A detailed list of volumes, white matter tracts, and white matter tract measures is provided in Supplementary Methods.

### 2.2 Analysis

#### 2.2.1 Workflow

We began with 19,408 subjects with completed CMR radiomics analysis of their short-axis imaging from the UK Biobank Imaging Extension. We downloaded and prepared the vascular risk factor, cognitive testing, brain imaging data, heart imaging, and covariates for these subjects (see Acquisition and Preparation). For each dataset separately, we dropped all subjects without complete data, merged all datasets, and selected only subjects without cardiovascular or brain disease (defined in Supplementary Methods). We then performed dimensionality reduction on each data type separately. We performed joint factorization of the heart and brain imaging data. We regressed out imaging confounders from the latent factors (Supplementary Methods). We merged the latent factors and performed all downstream analyses. We corrected all comparisons for multiple hypothesis testing with a Benjamini-Hochberg False Discovery Rate (BH-FDR) correction. Entire pipeline with number of subjects retained at each step reported in Supplementary Figure 1 and population statistics are reported in Supplementary Table 1. For every analysis, we present both raw and deconfounded results as paired Supplementary Tables, but we only discuss deconfounded results in the text. All code was open-sourced, see Data and Code Availability; the list of packages and settings used is in Supplementary Methods.

#### 2.2.2 Dimensionality reduction

##### 2.2.2.1 Latent variables for vascular risk (gVRF)

First, we derived an aggregate measure of vascular risk for each individual, counting instances of diagnosis of hypertension, diabetes, or hypercholesterolaemia, having ever smoked, having a BMI > 25, and having a high WHR (>0.85 for females and >0.90 for males) (Cox, Lyall, et al., 2019; Hamer & Batty, 2019). This factor is useful for clinical translation and also defining simple high- and low-risk groups.

We derived an additional latent factor of general vascular risk (gVRF) following prior work in this and other cohorts, using confirmatory factor analysis (CFA) in structural equation modelling (Cox, Lyall, et al., 2019; Wardlaw et al., 2014). This latent measure captures the tendency for VRFs to co-occur. gVRF was derived from smoking pack years, diastolic and systolic blood pressure, BMI, WHR, diagnosis of hypertension, diabetes, and hypercholesterolaemia. The model fit the data well, though loadings were inconsistent (range 0.189–0.745), with the factor more strongly loaded towards BMI and WHR (Supplementary Fig. 2, Supplementary Table 2). Because the aggregate measure of vascular risk relies on arbitrary cutoffs and there is high correlation between the aggregate measure and gVRF, we focus on gVRF in our discussion of all mediation analyses.

##### 2.2.2.2 Latent variables for cognitive function (general intelligence, g)

As previously reported (Lyall et al., 2017), we performed a CFA of the four cognitive tests. We hypothesised that the four tests would correlate moderately-highly (with intercorrelations of r > 0.40) and would form a single latent general factor (labelled g in prior literature) across the four tests with good fit to the data (Cox, Ritchie, et al., 2019; Deary et al., 2010; Lyall et al., 2016). We found this to be the case (Supplementary Fig. 3, Supplementary Table 3).

##### 2.2.2.3 Latent variables for heart structure

Since principal component analysis (PCA) is commonly used in radiomics to extract lower dimensional representations of the data (Antonopoulos et al., 2021; Raisi-Estabragh et al., 2020; Truhn et al., 2019; Zhang et al., 2017), we performed PCA on the z-scored radiomics. We chose the number of principal components using cross-validation, detailed in the Supplementary Methods. We kept the first three unrotated PCs (Supplementary Fig. 4, Supplementary Table 4). We extracted the scores of these components for each subject and used them for downstream analyses.

##### 2.2.2.4 Latent variables for brain structure

We isolated brain volume (“atrophy” after controlling for head size), grey matter volume, and total white matter hyperintensity volume (Cox, Lyall, et al., 2019). Latent measures of general white matter fractional anisotropy (gFA) and mean diffusivity (gMD) were derived using CFA, as previously reported in this cohort (Cox, Lyall, et al., 2019; Cox et al., 2016). The CFA models fit well with the lowest loadings for the corticospinal tracts and cingulate gyri and the highest loadings for the thalamic radiata and fasciculi (Supplementary Fig. 5, Supplementary Table 5).

Since principal component analysis has been used to capture variation in brain imaging in previous work and since we are using it to summarise the heart imaging in this work (Alfaro-Almagro et al., 2021; Elliott et al., 2018; Liang et al., 2021; Miller et al., 2016), we also computed PCA over all z-scored brain IDPs and selected the number of principal components to retain as before (Supplementary Methods). We kept three PCs (Supplementary Fig. 6, Supplementary Table 6). We extracted their scores for each subject and utilised them in downstream analyses. Because both CFA and PCA are widely used in the field and there is no definitive reason for preferring one to the other, we will include both in all downstream analyses to ensure that our findings are not dependent on the method of latent feature definition (John et al., 2012).

##### 2.2.2.5 Joint heart-brain factor analysis

Along with the factor analysis of the individual datasets described above, we also sought to derive latent factors that captured the main modes of correlated variation between heart and brain structural imaging. That is, we aimed to identify components of brain structure and components of heart structure that were maximally correlated. Through canonical correlation analysis (CCA) on the z-scored heart radiomics and brain IDPs, we derived 10 modes (Miller et al., 2016). Each mode consists of two components: (1) a linear combination of heart radiomics features and (2) a separate linear combination of brain IDPs that have highly similar variation in the population. The modes are ranked by the amount of correlation between the heart and brain components. We chose the number of modes to keep via cross-validation (Supplementary Methods), kept three modes (Supplementary Fig. 7, Supplementary Table 7), extracted the component scores for each subject in each dataset, and used them in downstream analyses.

#### 2.2.3 Descriptive statistics and associations

We conducted descriptive analyses, testing the association of age and sex with all of our latent variables using linear regression. We then examined the pairwise linear association between all latent variables by linearly modelling each latent variable as a function of sex, age, and each other latent variable. See Supplementary Methods for modelling details and how additional R^2 is computed. Results are reported for both raw and deconfounded imaging latents.

#### 2.2.4 Propensity score matching

Since all other analyses are performed on corrected, standardised, and latent measures of the data, we performed propensity score matching to yield real-units measurements of the differences between subjects with and without VRFs. We matched subjects with four or more VRFs with their nearest neighbour with no VRFs, requiring an exact match for sex (Supplementary Methods). We then performed repeated t-tests to compare the cognitive exam performance, CMR measures, and brain IDPs of the matched groups of subjects.

#### 2.2.5 Mediation modelling

To measure how well heart and brain structural features explain the VRF-CF association, we perform a series of mediation analyses (Bai et al., 2020; Ferguson et al., 2020; McCormick et al., 2022; Wardlaw et al., 2014). This method allows us to directly quantify the degree to which any identified associations between vascular risk and cognitive function are accounted for by brain or heart-based measures. The primary outcome is therefore the % of the gVRF-g association that is mediated when brain/heart measures are included in the model. In more complex models with more than one mediator, one can also identify which mediator is contributing the largest unique mediating effect. Thus, these analyses offer an elegant quantitative solution for identification of important heart and brain biomarkers underpinning VRF-cognitive associations. We report a more complete description of the mediation model in the Supplementary Methods.

We first performed mediation models on solely the latent representations of each data set. We found the association between gVRF and g and then modelled how well each imaging latent variable mediated this association (more details in Supplementary Methods). At first, we only modelled one imaging latent at a time, calling this the “Latent Single Mediation Model.” Then we performed both parallel and sequential multiple mediation analyses, fixing heart PC2 as the first mediator and then adding brain latents as the second mediator, called “Latent Multiple Mediation Model.” Next, we replaced the gVRF-g association with pairs of individual VRFs and cognitive exams, testing imaging latents one at a time again, calling this “Latent Single Mediation Modelling of VRF-Cognitive Pairs.” Given the high association between the VRFs (Supplementary Fig. 2, Supplementary Table 2), we control each VRF-exam association for all other VRFs to identify unique associations between each VRF and cognitive exam.

To explore the role of individual imaging features in explaining the association between VRFs and CF, we returned to the gVRF-g association and performed mediation modelling for each imaging feature individually, calling this the “Individual Feature Single Mediation Model.” We perform modelling as described in Supplementary Methods and always control for age and sex.

Given that all latent measures across domains (vascular risk, heart, brain and cognitive) were standardised, reported coefficients are standardised regression coefficients (i.e., β range [-1,1]) throughout, allowing direct comparison of effect magnitudes across modalities.

## 4 Results

### 4.1 Quantifying heart brain axes

**Fig. 1.**
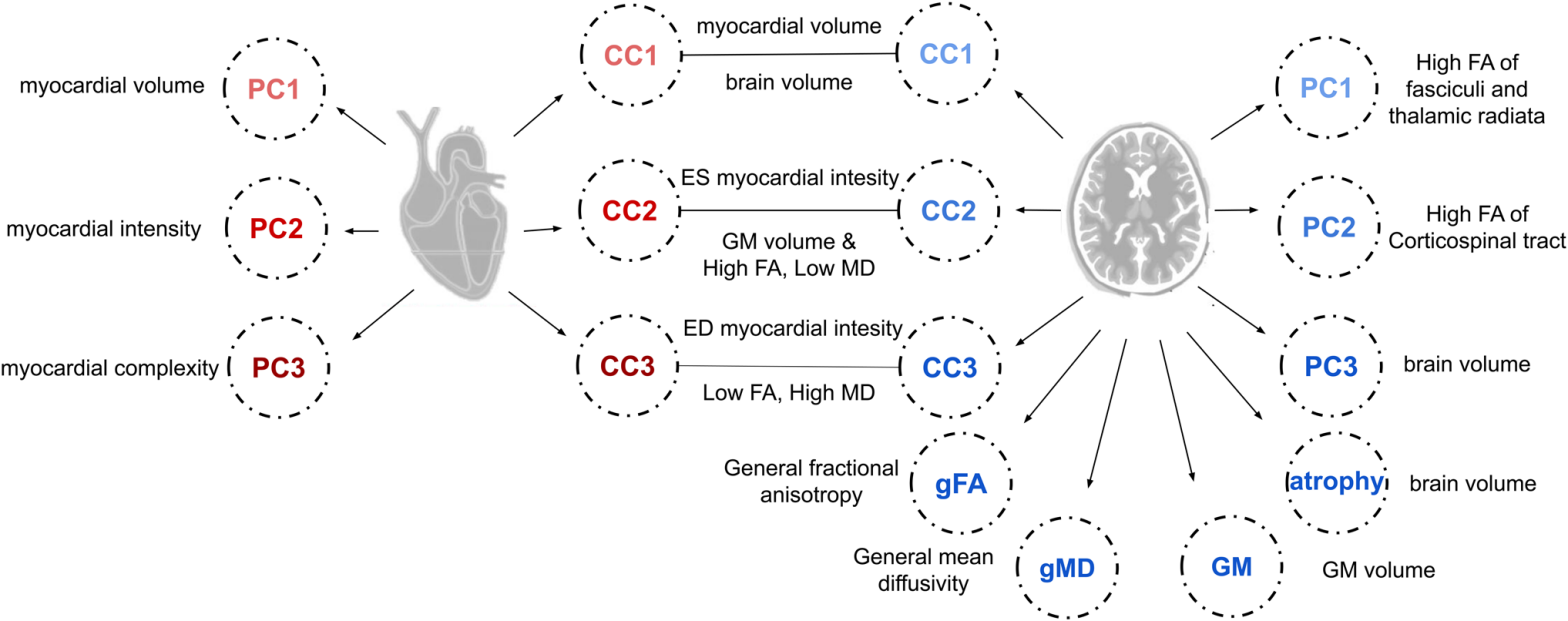
Latent factors. A schematic illustration is provided of all of the extracted latent factors and a simple interpretation of their meaning is given. The loadings for all the factors can be found in the Supplementary Tables and more detailed interpretations of the meaning of each factor can be found in the Supplementary Methods.

After our data preparation pipeline yielded 11,962 subjects (Supplementary Fig. 1, Supplementary Table 1), we quantified key axes of variation in all four of our datasets. We extracted latent measures of vascular risk (gVRF), cognitive function (g), and brain structure as reported previously (see Methods) (Cox, Lyall, et al., 2019; Cox, Ritchie, et al., 2019; Ferguson et al., 2020; Lyall et al., 2016, 2017). Along with traditional measures, we performed PCA of heart and brain imaging separately and a novel CCA to capture correlated variability in heart and brain structure (Fig. 1). For cardiac radiomics, the first three PCs explain 25, 20, and 12% of the variance and represent myocardial size, intensity, and textural complexity respectively (Supplementary Fig. 4, Supplementary Table 4). For brain MRI indices, the first three PCs explain 30, 12, and 8% of the variance and represent high FA and low MD of the fasciculi and thalamic radiata, high FA and low MD of the corticospinal tract, and brain volume respectively (Supplementary Fig. 6, Supplementary Table 6). For the joint heart brain axes, the first three modes have a Pearson correlation of 0.71, 0.48, and 0.32 respectively (Supplementary Fig. 7, Supplementary Table 7). Based on the loadings, we interpreted that the heart brain axes correspond to (1) heart and brain volume, (2) end-systolic myocardial intensity, grey matter and thalamic volume, and thalamic radiation WM integrity, and (3) end-diastolic myocardial intensity and low FA and high MD of many tracts (more details in Supplementary Methods).

### 4.2 Descriptive statistics

Nearly all latent variables have a significant association with age and sex (Supplementary Fig. 8, Supplementary Tables 8, 9). Older subjects show lower aggregate performance on cognitive exams (β = -0.183) and greater vascular risk (β = 0.171) (Cox, Lyall, et al., 2019; Lyall et al., 2017). Among the heart structural latents, old age associates with slightly greater myocardial volume (CMR PC1, β = 0.035), lower myocardial intensity (PC2, β = -0.173), and lower myocardial textural complexity (PC3, β = -0.109) (Rouch et al., 2022).

Among the brain structural latents, old age associates with lower total and grey matter volume, lower white matter integrity (β range -0.363 to -0.249), and greater white matter hyperintensity volume (β = 0.353). Age also strongly negatively associates with the components of the second CCA mode, representing lower myocardial intensity, grey matter and thalamic volume, and thalamic white matter integrity (β range -0.591 to -0.441).

### 4.3 Associations between vascular risk, heart, brain, and cognition

**Fig. 2.**
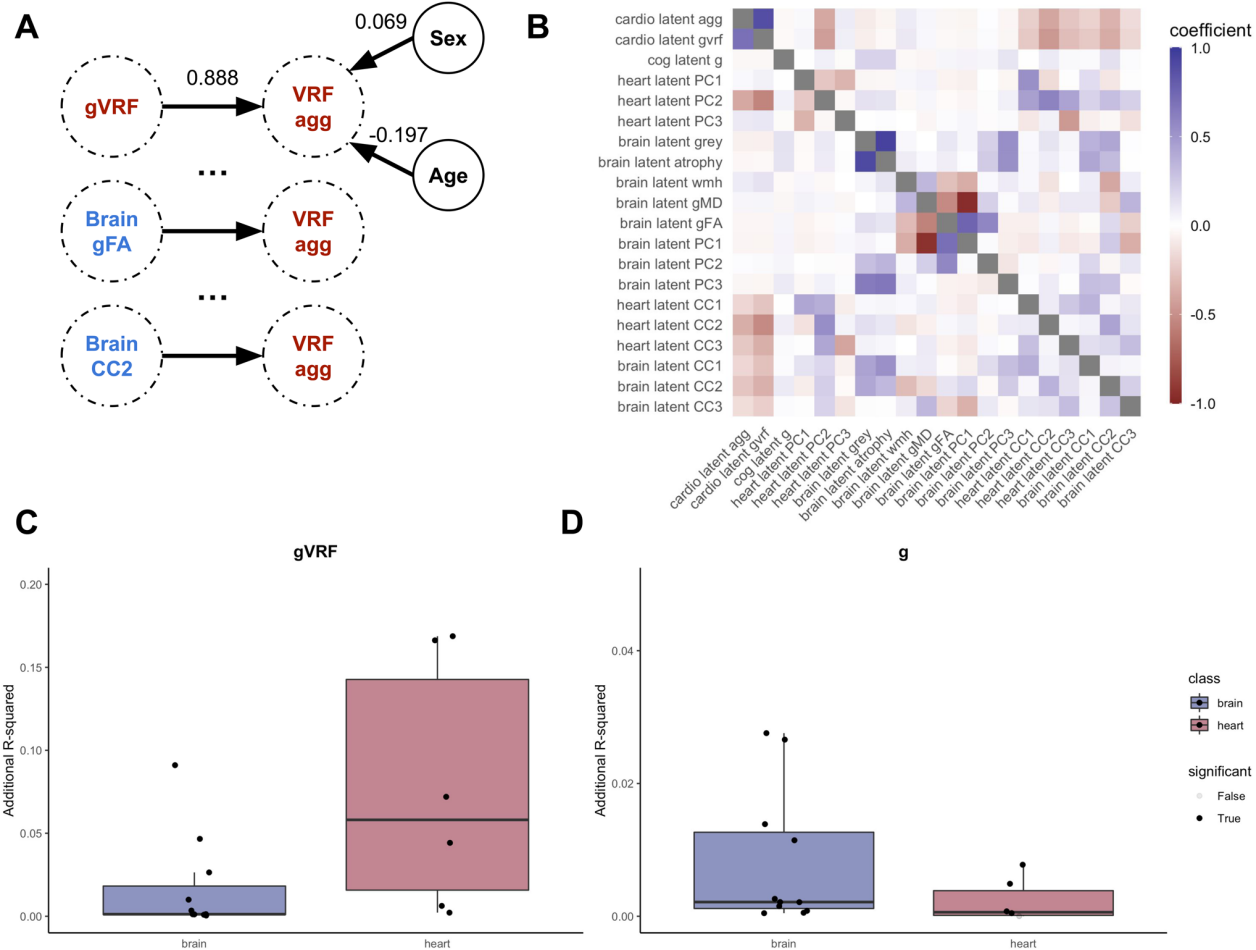
Pairwise latent associations. We modelled the association between every pair of latent variables. (A) A schematic diagram of the modelling process. Every latent variable (e.g., VRF agg) is linearly modelled as a function of another latent variable (e.g., gVRF), sex, and age. The derived coefficients for the example first model are illustrated. We repeat this for every variable, and the coefficients from these analyses compose the first row of the adjacent heatmap. (B) Heatmap of standardised coefficients from all 342 separate pairwise linear models. Each row lists the dependent variable, and each column lists the independent variable in the linear models. (C) With gVRF set as the dependent variable, we compare the R-squared of the linear model for each latent grouped by whether it was derived from the heart or brain imaging. (D) With g set as the dependent variable, we compare the R-squared of the linear model for each latent grouped by whether it was derived from the heart or brain imaging. All model estimates are reported in Supplementary Table 11.

Associations among each pair of latent variables were modelled separately, controlling for age and sex (Fig. 2, Supplementary Tables 10, 11). There is a small but significant negative association between gVRF and g (β = -0.036), consistent with prior reports (Ferguson et al., 2020; Lyall et al., 2017). Many imaging latents across heart and brain associate with both greater gVRF and lower g: lower latent myocardial intensity, lower total and grey matter volume, lower white matter tract integrity, and greater white matter hyperintensity volume (Fig. 2, Supplementary Table 11).

All of the heart PCs explained at least an order of magnitude more variance in gVRF (additional R^2^: 0.002–0.166) than in g (aR^2^: 0–0.004) (Fig. 2). Similarly, the brain volume latents (atrophy, grey matter volume, PC3) explained at least an order of magnitude greater variance in g (aR^2^: 0.012–0.027) than in gVRF (aR^2^: 0.0006–0.003). Interestingly, the second joint factor (CC2) explains more similar amounts of variance in both g (aR^2^: 0.009–0.013) and gVRF (aR^2^: 0.089–0.164), and it explains at least an order of magnitude more variance in both g and gVRF than the white matter latents. This suggests that leveraging information from both heart and brain structure is useful in deriving factors that explain a relatively large and equal amount of variance in both vascular risk and cognitive function.

### 4.3 Matched analysis

Aware that the latent measures are all in arbitrary units, we used propensity score matching to provide more practically interpretable information on how those with high and low vascular risk differ across heart, brain, and cognitive measures, in native units. We assembled two groups of 425 subjects matched by sex, age, head size, and BSA (Supplementary Table 12). On average, when compared to matched individuals with no VRFs, subjects with 4 or more VRFs have 13.09 mL (8.29%) lower LVEDV, 7.56 mL (11.50%) lower LVESV, and 5.52 mL (5.99%) lower LVSV. Consistent with mild ventricular hypertrophy, the subjects with 4 or more VRFs have 1.51% (2.58%) greater ejection fraction. We find lower average intensities of the myocardium in end-systole (23.53%) and -diastole (19.65%). We also find greater uniformity of the myocardial tissue appearance (5.25–8.37%). These subjects also have 14,357 mm^3^ (2.31%) less grey matter volume and additionally lower subcortical volumes. They have greater white matter hyperintensity volume (62.34%). They also have lower FA in many tracts (range 0.96% and 1.92%). Compared to matched healthy controls, subjects with 4 or more VRFs also score on average 0.48 (6.67%) fewer points on verbal-numerical reasoning. These subjects also have notable differences in their latent measures, like greater myocardial size, poorer white matter tracts, and lower second heart-brain axis (myocardial intensity, grey matter volume, thalamic WM tract integrity). Simply summing risk factors correlates with gVRF (Fig. 2, Supplementary Table 11), and this matched analysis shows that the sum manifests with clinically observable phenotypes in heart imaging, brain imaging, and cognitive exam performance.

### 4.4 Latent single mediation modelling

**Fig. 3.**
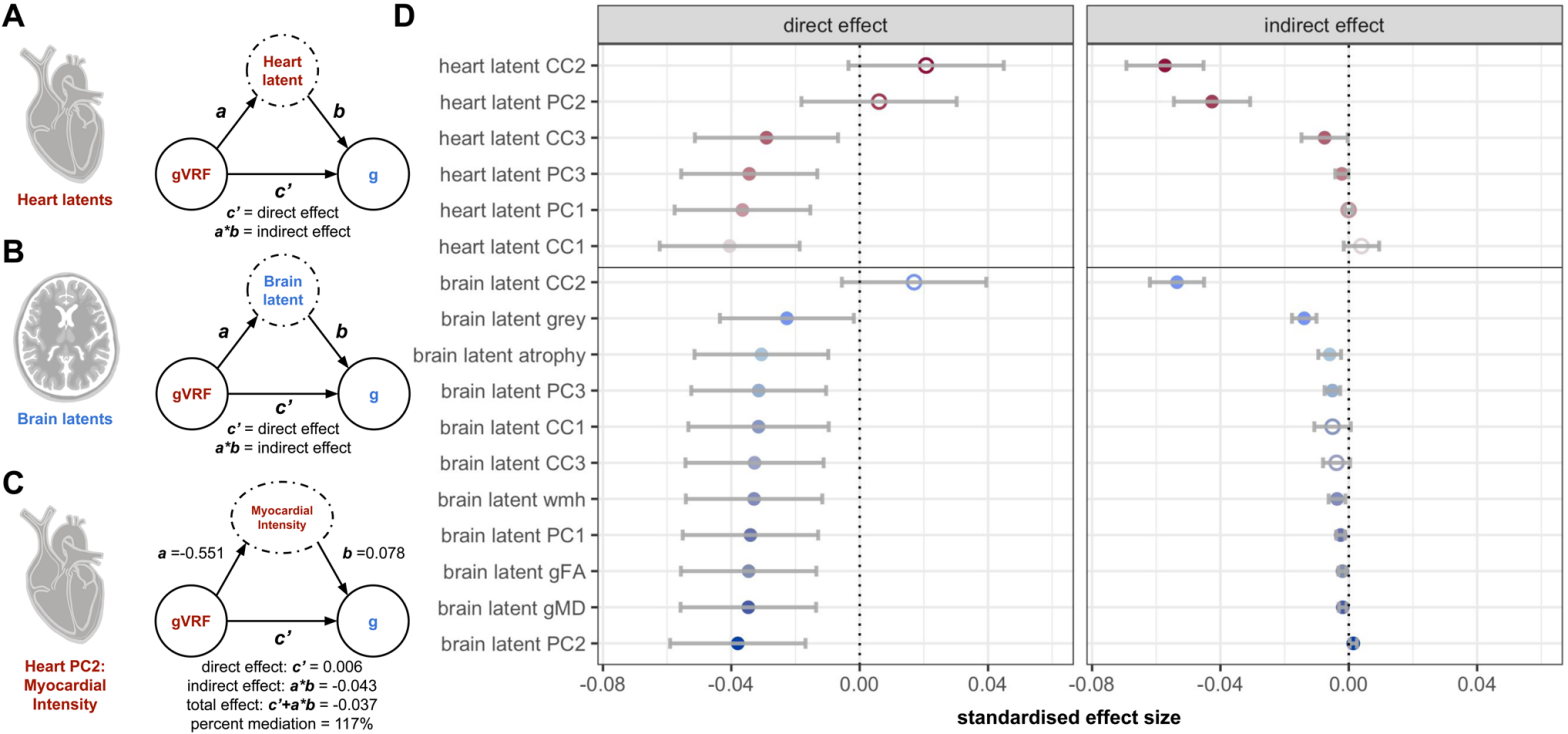
Latent single mediation modelling. We performed serial mediation modelling of the gVRF-g association, testing each imaging latent as a potential mediator. (A) Schematic for the CMR radiomics modelling procedure. gVRF and g were maintained as the known association, and we iterated over all CMR imaging latent factors. Equations demonstrate the derivation of the direct and indirect effect. (B) Schematic for the brain MRI modelling procedure. (C) Example computation of the measured effects. Confidence intervals reported in Supplementary Table 14. (D) The estimates for the direct and indirect effects for all potential mediators, sorted by indirect effect size, closed circles are significant (p < 0.05) and open are not. Error bars derived from bootstrapping (see Supplementary Methods).

Initially, we asked the degree to which each brain or heart measure, in isolation, mediates the association between vascular risk and CF. Results are presented in Figure 3, Supplementary Tables 13, 14. Consistent with prior reports, measures of brain structure—irrespective of how they were measured—only modestly mediated the association (4.97–38.12%), with white matter measures being the smallest, but still significant, mediators. However, latent myocardial intensity (heart PC2) and the heart-brain axis capturing myocardial intensity, grey matter volume, and thalamic white matter integrity (CC2) all completely mediate the gVRF-g association (117%-150%; attenuated to be indistinguishable from *β* = 0 in each case). For example, one standard deviation (SD) lower gVRF associates with 0.55 standard deviation lower latent myocardial intensity. This 0.55 SD lower intensity associates with 0.043 SD lower cognitive function.

As a control, we address two possible counterarguments: (1) that the BMI-cognitive function association is the only VRF well explained by myocardial intensity and (2) that latent myocardial intensity is just a proxy for myocardial size. First, since gVRF most strongly weights BMI and WHR (Supplementary Table 2), it is possible that the gVRF-g association is driven primarily by BMI and that latent myocardial intensity only mediates the BMI-g association. However, covarying for BMI partly attenuated, but did not remove, myocardial intensity’s mediation of the gVRF-g association (40.18%) (Supplementary Table 15). Second, since latent myocardial intensity and myocardial volume are associated (Supplementary Table 11), it is possible that latent myocardial intensity is just a measure of myocardial size not well adjusted by regressing out BSA. However, we show that latent myocardial intensity associates with BMI independent of body and myocardial size (Supplementary Table 15). Therefore, latent myocardial intensity’s mediation of the gVRF-g association is not just explained by the BMI-g association and, furthermore, the BMI-latent myocardial intensity association is not just due to the myocardium being larger.

### 4.5 Latent multiple mediation modelling

**Fig. 4.**
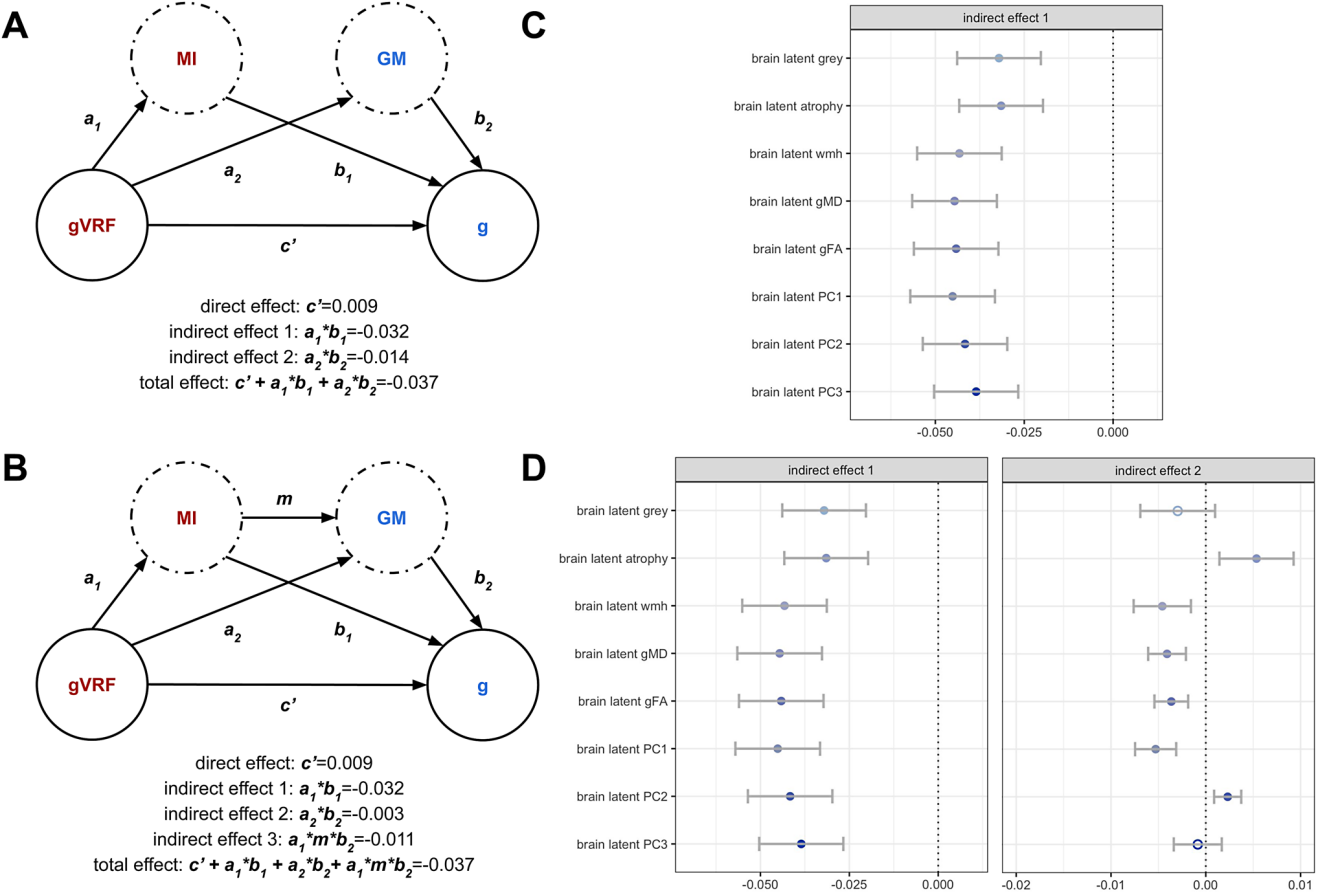
Latent multiple mediation modelling. We performed both parallel and sequential multiple mediation modelling of the gVRF-g association, including heart PC2 as the first mediator and then considering all brain latents as second mediators. (A) Schematic of the parallel modelling procedure with a single direct effect and two indirect effects, one for each potential mediator. We list values from an example mediation effect in which grey matter volume was the second mediator. Confidence intervals are reported in Supplementary Table 17. The direct effect is fixed for all mediators at 0.009. (B) Analogous schematic for the sequential modelling procedure. Values reported from an example model with grey matter as the second mediator. Full data are provided in Supplementary Table 19. (C) A bar chart of the estimates for the indirect effect for heart PC2 when each brain latent was used, closed is significant (p < 0.05) and open is not. (D) A bar chart of the estimates for the indirect effect for each brain latent when using either parallel (left) or sequential (right) mediation.

The structural functional model argues that heart structural variation impacts cognitive function via its impact on brain structure. To model this within our data, we constructed two related multiple mediation models (Fig. 4). In the first model, we performed “parallel” multiple mediation that does not account for the heart-brain association. In the second model, we performed “sequential” multiple mediation that does account for the heart-brain association.

We performed this analysis for latent myocardial intensity paired with every brain latent since latent myocardial intensity was the only significant heart mediator. Comparing the heart indirect effects between the single mediation (Fig. 3) and the parallel multiple mediation (Fig. 4C, Supplementary Tables 16, 17) allows us to assess the impact of brain structure on the heart-cognitive function association. The heart indirect effect slightly decreases when accounting for brain volumes but not brain white matter measures. Therefore, brain volume variation can explain some but not all of the association between latent myocardial intensity and cognitive function. Comparing the brain indirect effects between the parallel and sequential mediation allows assessment of the impact of latent myocardial intensity on the VRF-brain association. In this case, the brain volume measure indirect effects go to zero but the white matter indirect effects do not decrease (Fig. 4D, Supplementary Tables 18, 19). Thus, latent myocardial intensity variation can explain all of the association between VRFs and brain volume but not VRFs and white matter intensity.

### 4.6 Latent single mediation modelling of individual VRF-cognitive pairs

Recent work has noted the potential for spurious mediations when modelling with composite measures; for example, false “interrupted mediators” occur when one component of a composite mediator associates with only the causal variable and one component associates with only the outcome. To confirm that our results are robust to this concern, we spend the next two sections analysing mediation using individual measures. We first consider pairs of individual VRFs and cognitive exams (Supplementary Fig. 9, Supplementary Tables 20, 21). We found that pack years and VNR (β = -0.028), WHR and VNR (β = -0.061), and WHR and RT (β = 0.032) all had independent associations in the expected directions. Brain volumetric latents most strongly mediated the pack year–VNR association (12.11–47.64%), while myocardial intensity associated latents most strongly mediated the WHR-VNR association (27.33–42.76%). The myocardial intensity features are also the only significant mediators of the WHR-RT association (34.75–49.52%). Likely because they capture some relevant variation in brain volumes, white matter tracts, and myocardial intensity, the components of the second joint factor strongly mediate both the pack-year and WHR cognitive exam associations (21.63–49.52%).

### 4.7 Individual feature mediation modelling

**Fig. 5.**
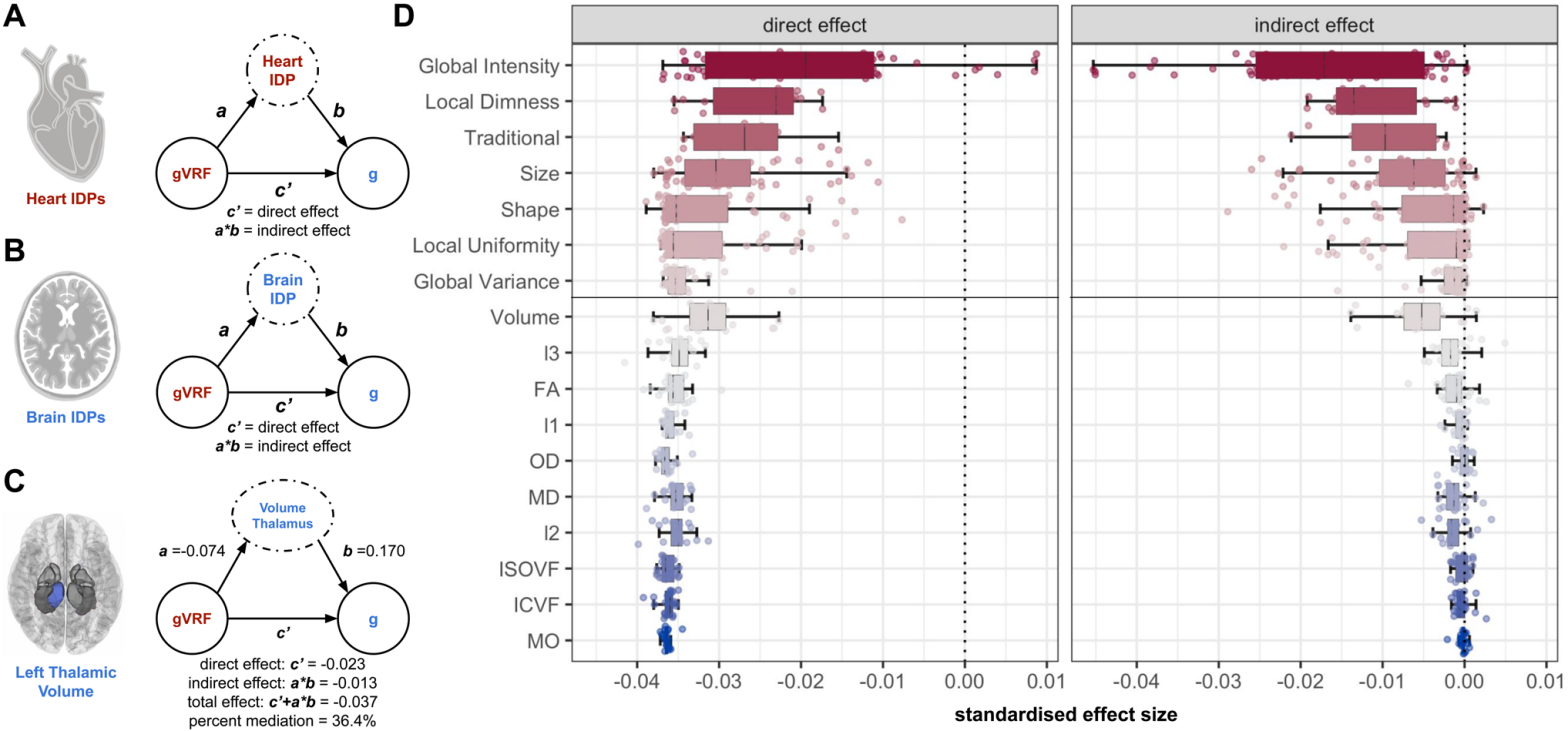
Individual feature single mediation modelling. We performed serial mediation modelling for all individual imaging features. (A) Schematic for the modelling procedure for CMR IDPs. Same as Figure 3, except all potential mediators are now individual features (IDPs) instead of latent variables. (B) Schematic for Brain IDPs. (C) Example mediation model for an individual feature with the left thalamic volume (Volume Thalamus) as a potential mediator. Confidence intervals are reported in Supplementary Table 25. (D) Direct and indirect effects for all tested CMR radiomics grouped by cluster and brain MRI IDPs grouped by their feature type. For visualisation, we grouped the brain IDPs by their IDP categories and the CMR radiomics by previously reported clusters extracted from imaging of healthy individuals (Raisi-Estabragh, Jaggi, et al., 2021). We also include conventional CMR indices as a separate cluster.

Continuing our controls for spurious mediation from composite measures, we next turn to individual imaging measures. Although the latent imaging features capture large amounts of the variance in the imaging datasets (Supplementary Figs. 4, 6), each imaging dataset contains many features and much variance beyond the latents used in the previous analyses. To offer a comprehensive picture of how heart and brain structure mediate the gVRF-g association, we perform single mediation analysis for every individual imaging feature (Fig. 5, Supplementary Tables 22, 23, 24, 25).

As expected, many individual features associated with myocardial intensity show complete mediation (Fig. 5, Supplementary Tables 22, 23). However, a number of CMR measures showed mediating effects that were previously difficult to appreciate via latent modelling. While the latent measure of myocardial volume did not mediate the association (Fig. 3), both the right and left ventricular volumes partially mediated the association (32.5–61.1%). Although the latent measure of myocardial tissue complexity was just below significance (Supplementary Table 14), some measures of local nonuniformity and local homogeneity partially mediated the association (32.5–48.5%). Greater local nonuniformity associated with lower vascular risk (β = -0.347– -0.284) and greater cognitive function (β = 0.051–0.054), and measures of local homogeneity show the opposite associations (Supplementary Table 23).

Compared to the heart, the brain IDPs show an order of magnitude lower indirect effects and proportionally lower percent mediation (Fig. 5, Supplementary Tables 24, 25). Of the brain IDPs, volumes have the largest mediating effect, particularly grey matter (38.1%) and thalamic volume (35.9–36.4%). The largest white matter microstructural mediating effects are from the thalamic radiation tracts (Supplementary Fig. 10). For example, MD of all the thalamic radiation tracts significantly mediates the association (5.17–8.26%), and the FA of the left posterior thalamic radiation tract has the greatest mediation of all the white matter microstructural mediating effects (18.3%).

For both the VRF-cognitive exam pairs and the individual mediators, the existence of significant mediation for individual features argues against potential spurious mediation due to composite measures (McCormick et al., 2022).

Lastly, we considered whether an expanded set of CMR indices, including myocardial strain, myocardial thickness, aortic dilation, and other advanced shape-based features, could mediate the vascular risk–cognitive function association (Bai et al., 2020; Zhao et al., 2023). After controlling for body surface area, none of these advanced shape measures are significant mediators of the vascular risk–cognitive function association (Supplementary Table 26).

## 5 Discussion

### 5.1 Interpretation

This study supports the structural-functional model for the link between vascular risk and cognitive function. Many heart and brain structural measures separately mediate the vascular risk–cognitive function association (Figs. 3, 5). Despite these initial results, it was still possible that the mediating measures from the heart and brain shared no inter-associations. This would violate the structural-functional model’s claim that vascular risk causes vascular and cardiac remodelling, which, in turn, causes cerebral damage. Our results argue against this possible negative result in three ways. First, we find significant associations among the separate heart and brain mediators, like between heart PC2 and grey matter volume (Fig. 2). Second, we find that these associated mediators have partially overlapping mediating effects (Fig. 4). Third, we find that one of the major axes of covariance between heart and brain structure (CC2) significantly mediated the VRF-CF association (Fig. 3). Therefore, the heart and brain do indeed share mediating effects, indicating that their variation may be linked via the structural-functional model.

Our results also complicate the structural functional model. When comparing the multiple mediation models (Fig. 4), we found that latent myocardial intensity variation can fully explain the VRF-grey matter association, but grey matter variation cannot fully explain the latent myocardial intensity–cognitive function association. This suggests that heart structural variation can associate with cognitive function in ways independent of brain structural variation. This violation of the structural functional model could be explained by brain changes not well captured by our metrics (e.g., smaller cortical grey matter changes). Additionally, we found that latent myocardial intensity cannot explain the VRF-white matter integrity association (Fig. 4). Therefore, the brain associates with risk factors in manners independent of cardiac variation. Mechanisms for this break in the model could be explained by direct impact of metabolic hormonal dysregulation on the brain or brain vasculature, without affecting heart structure.

Consistent with prior reports, considering brain structural measures alone only accounted for a minority of the VRF-CF association (Ferguson et al., 2020). Although important features from these brain latents all have relatively large coefficients in the second heart-brain CCA mode (Supplementary Table 7), these latents alone show much smaller indirect effects than the second heart-brain CCA mode (Supplementary Table 14). The strong alignment of the second CCA mode with the VRF-CF association suggests that leveraging the association between heart and brain structure is informative to deriving a brain imaging latent factor that associates with both vascular risk and cognitive decline. In other words, without considering vascular risk or cognitive function in their derivation, one can discover brain biomarkers that better explain the VRF-CF association by using the heart-brain structural association.

Focusing on the brain structures identified, this work unifies separate findings that have shown that lower grey matter and thalamic volume associates with greater vascular risk and lower cognitive function (Bai et al., 2020; Cox, Lyall, et al., 2019; Cox, Ritchie, et al., 2019; Ferguson et al., 2020). Furthermore, this work supports the association of deteriorating thalamic tract white matter microstructure with elevated vascular risk and poorer cognitive function (Cox, Lyall, et al., 2019; Cox, Ritchie, et al., 2019). Previous work has argued that the thalamus is both central to integrative signalling in the brain and potentially susceptible to changes in cerebrovascular perfusion (Bohlken et al., 2014; Cox et al., 2016; Payabvash et al., 2011; Rikhye et al., 2018). Crucially, this works links variation in these structures to myocardial intensity. Why exactly thalamic volume and thalamic white matter integrity associate with myocardial intensity is still unknown and will be of interest in future work.

Our analyses of individual VRFs and cognitive exams revealed subtle trends not apparent in our more global/latent results, where brain and heart had differential importance. For example, whereas brain volumes more strongly mediate the pack year–VNR association than the WHR-VNR association, myocardial intensity exhibited the reverse pattern (Supplementary Fig. 9, Supplementary Table 21). This result highlights the utility of a comparative approach between heart and brain structural variation. However, the individual VRF cognitive exam analysis also revealed the complexity in some of these phenotypes, replicating a previous finding of a positive association between BMI and visual memory (Supplementary Table 21) (Ferguson et al., 2020).

Beyond supporting findings from the latent analysis, the individual gVRF-g mediation analysis of imaging features revealed that lower right and left ventricular volume for body size associates with greater vascular risk and lower cognitive function (Fig. 5, Supplementary Table 23). This result could point to a simple mechanistic step in the structural-functional hypothesis in which lower stroke volume for body size decreases cerebral perfusion (Rouch et al., 2022). Analysis of the individual brain features highlights grey matter, some subcortical volumes, and thalamic white matter tract measures as most mediating the gVRF-g association (Fig. 5, Supplementary Table 25). This provides independent support from the joint analysis that these specific brain structures are key to the heart-brain axis.

Lower myocardial intensity has previously been associated with specific vascular risk factors and greater red meat consumption, and, here, we quantify its association with both greater aggregate vascular risk and lower cognitive function (Cetin et al., 2020; Raisi-Estabragh, Jaggi, et al., 2021; Raisi-Estabragh, McCracken, et al., 2021). Lower myocardial intensity strongly associates with higher vascular risk in a manner not explained by body or heart size (Supplementary Table 15). Additionally, myocardial thickness cannot explain the vascular risk–cognitive function association (Supplementary Table 26). These findings suggest that myocardial intensity is a myocardial size-independent biomarker for vascular risk. Lower myocardial intensity could have several biological interpretations. Previous imaging studies have detected myocardial fibrosis in cohorts of patients with vascular risk factors, suggesting that the low intensity features common to vascular risk and cognitive decline could be signs of a common myocardial fibrotic pathology driven by vascular risk factors (Mavrogeni et al., 2017; Ng et al., 2012; Turkbey et al., 2015). We also found some mediation via greater myocardial textural uniformity (Supplementary Table 23), which could also associate with the speculated fibrosis. Alternatively, since blood appears bright in CMR, lower myocardial intensity could suggest lower myocardial perfusion, compromising cardiac function and cerebral circulation. These results motivate further work to confirm these hypotheses through detailed imaging and tissue pathology.

### 5.2 Limitations

Although this study uses an exceptionally large dataset of adults across a wide range of middle- and older ages, this work does not analyse longitudinal data. Therefore, we cannot disambiguate whether cardiovascular risk is causing decreased cognitive function, lower cognitive function is causing increased cardiovascular risk, or some mix of both effects. However, numerous longitudinal studies in other cohorts support that cardiovascular risk associates with accelerated cognitive decline (Knopman et al., 2001; Rusanen et al., 2014; Samieri et al., 2018; SPRINT MIND Investigators for the SPRINT Research Group et al., 2019; W. Wang et al., 2022; Yaffe et al., 2014). Furthermore, without longitudinal imaging, we cannot assess the temporal relationship between cardiac and brain imaging phenotypes, vascular risk, and cognitive function. However, we argue that our results still offer novel cross-sectional support for the structural-functional model linking elevated vascular risk and poorer cognitive function.

In this work, we focus on the structural functional model linking vascular risk and cognitive function. Importantly, the VRF-CF association could be equally well explained by unmeasured mechanisms (e.g., metabolic hormonal dysregulation could directly impact neuronal function) (Benedict et al., 2007) or by reverse causation (e.g., poor cognitive function could decrease healthy lifestyle maintenance) (Batty et al., 2007; Calvin et al., 2011). Testing these hypotheses adequately would require longitudinal and biochemical data not yet available via the UK Biobank (Jensen et al., 2023; M. Wang et al., 2016). The UK Biobank does offer numerous measurements of possible confounders (socioeconomic status, geography, lifestyle, etc.) that could be used to rule out potential sources of error in our estimates. Although this study attempts to control for several well-studied sources of biometric and imaging confounding, future work could survey the wide array of possible confounders to refine our initial estimates.

We do not adjust for ethnicity in this study due to the low numbers of non-White British participants and the heterogeneity of those minority participants (Supplementary Table 1). Because the UK Biobank represents a relatively homogenous, well-educated, higher socioeconomic status, and predominantly Caucasian population, we emphasise the lack of generalizability of our findings to other populations. Neurovascular disease may differ significantly across populations of different ancestries and economies. Increased longitudinal biobanking of more diverse populations will be crucial to extending our findings to a more representative sample of the global population (Prictor et al., 2018; Ricard et al., 2023).

Whereas some have questioned the reliability of the UK Biobank cognitive exams (Lyall et al., 2016), recent work has supported their validity and psychometric properties (Fawns-Ritchie & Deary, 2020). Additionally, as reported in previous work, the effect sizes for the association between individual VRFs and cognitive exams is small, and we find no unique association for many VRFs and at least two associations pointing in the “opposite direction” as hypothesised (Supplementary Fig. 9, Supplementary Tables 21) (Lyall et al., 2017). Results from the full UK Biobank study suggest that large studies are needed to consistently detect these small effects and future increases to the imaging subset will help refine our results (Ferguson et al., 2020; Newby et al., 2021). We argue that the approach implemented here, via obtaining a latent measure g, minimises the impact of individual exam variability by obtaining an estimate of a robust, replicable, and test-invariant cognitive construct (Cox, Ritchie, et al., 2019; Fawns-Ritchie & Deary, 2020; Lyall et al., 2016).

## 6 Conclusion

The structural-functional model explaining the VRF-CF association rests on the argument that vascular risk drives changes in cardiovascular structure that lead to alterations in brain structure that lead to cognitive decline. Definitive support for the causal sequence of this model would require experimental or longitudinal work. However, our models (using cross-sectional data) are consistent with the hypothesis that vascular risk-associated cognitive ageing associates with distinctive variation in cardiac and brain structure. This is the first large-scale work to show that there is correlated variance in both heart and brain structure that mediates the association between vascular risk and cognitive function, providing a more extensive multi-modal framework to important prior work (Bai et al., 2020; Cox, Lyall, et al., 2019; Cox, Ritchie, et al., 2019; Ferguson et al., 2020; Lyall et al., 2017; McCracken et al., 2021; Newby et al., 2021; Raisi-Estabragh, Jaggi, et al., 2021; Raisi-Estabragh, M’Charrak, et al., 2021). One of the many hypotheses generated from analysing these data together is the identification of a key link to explain: how myocardial hypointensity could associate with cerebrovascular hypoperfusion impacting particular subcortical structures, like the thalamus.

## Supplementary Material

Supplementary Material

Supplementary Material

## Data Availability

UK Biobank Data are available via application. All code open-sourced here: https://github.com/akshay-jaggi/heart_brain_mediation.
