# Supplementary material for: A structural heart-brain axis mediates the association between cardiovascular risk and cognitive function
Source: Imaging Neurosci (Camb). 2024 Jan 25;2:imag-2-00063. doi: 10.1162/imag_a_00063 (PMC12224448; doi:10.1162/imag_a_00063)
Supplement: Supplementary Material [file imag_a_00063-supp.pdf]

Supplementary Methods

Acquisition and Processing

Vascular Risk Factors

Diabetes diagnosis was confirmed by combining data from ‘Diagnosed by doctor,’ self-report, ICD10 codes, first HES recorded occurrences, recorded use of insulin, and blood glycated haemoglobin levels (HbA1c). Hypercholesterolemia was confirmed by combining data from ICD10 codes, first HES recorded occurrences, recorded use of cholesterol lowering agents, and blood cholesterol levels. Hypertension was confirmed by combining data from self-report, ICD10 codes, first HES recorded occurrences, recorded use of blood pressure medications, and an HES physician diagnosis of hypertension. In all cases, if one or more of these measures was consistent with the diagnosis, we considered the individual as having the diagnosis. Exact list of UK Biobank field IDs found in prior publication (Raisi-Estabragh, Jaggi, et al., 2021).

Cardiac Imaging

The UK Biobank acquired all images according to predefined standardised operating procedures (Petersen et al., 2016; Raisi-Estabragh, Harvey, et al., 2021). The relevant imaging comprised of a complete short axis stack covering the left and right ventricles acquired at one slice per breath hold using balanced steady- state free precession (bSSFP) sequences. Typical acquisition parameters are as follows: TR/TE = 2.6.1.1 ms, flip angle 80 degrees, Grappa factor 2, voxel size 1.8mm × 1.8mm × 8mm. The actual temporal resolution of 32ms was interpolated to 50 phases per cardiac cycle (~20 ms). With the exception of distortion correction, no signal or image filtering was applied.

The first 5,000 UK Biobank CMR scans were manually segmented using CVI42 post-processing software (Version 5.1.1, Circle Cardiovascular Imaging Inc., Calgary, Canada). The analysis protocol has been previously published.

In brief, LV endocardial and epicardial borders were contoured in end-diastole and end-systole in the short axis stack images. End-diastole was defined as the first phase of the acquisition. End-systole was selected as the cardiac phase at which the mid-ventricular LV intra-cavity blood pool appeared smallest by visual inspection. The right ventricular (RV) endocardial borders were segmented in end-diastole and end-systole. This manual ground-truth dataset was used to develop a fully automated image segmentation algorithm applied to the rest of the available UK Biobank CMR studies (Attar et al., 2019; Bai et al., 2018; Petersen et al., 2017).

The segmentations from the short axis stack were then used to define three regions of interest for radiomics analysis: RV cavity, LV cavity, LV myocardium. Radiomics features are calculated from 3D volumes of these ROIs. On a high level, the shape features capture volume, maximal diameter, elongation, and related features of each structure; the intensity features capture global intensity statistics like mean MR intensity of each structure; and the texture features capture local patterns of MR intensity using methods like grey-level co-occurrence matrices. To reduce intensity level variations attributable to the acquisition process, we performed intensity normalisation of images through histogram matching, using as reference one of the studies from the dataset (Carré et al., 2020; van Timmeren et al., 2020). For grey level discretisation, we used a fixed bin width of 25 intensity values. Along with radiomics features, we also extracted traditional measures, including left ventricular end-systolic volume (LVESV), end-diastolic volume (LVEDV), stroke volume (LVSV), ejection fraction (LVEF), corresponding right-ventricular values (RVESV, RVEDV, RVSV, RVEF), and left ventricular mass (LVM).

Lastly, in a subset of our cohort consisting of 7118 individuals, we computed 78 features as prescribed in Bai, et al. (Bai et al., 2020). Specifically, we generated wall thickness features from short axis images, including global mean wall thickness and the mean wall thickness of 16 American Heart Association (AHA) segments (denoted as WT_AHA_1, …, WT_AHA_16). From long axis images, we generated LA maximum volume (LAV.max), LA minimum volume (LAV.min), LA stroke volume (LASV), LA ejection fraction (LAEF), RA maximum volume (RAV.max), RA minimum volume (RAV.min), RV stroke volume (RASV), and RA ejection fraction (RAEF). From aortic cine image features, we included AAo maximum area, AAo minimum area, AAo distensibility, DAo maximum area, DAo minimum area, and DAo distensibility. We computed circumferential and radial strains (Ecc and Err) globally and from

the 16 AHA segments. Lastly, we compute peak longitudinal strains (Ell) globally and from six segments of the myocardial contour based on the long axis images: basal septal, basal lateral, mid septal, mid lateral, apical septal, and apical lateral.

Brain Imaging

All brain MRI data were acquired on the same model of 3T Siemens Skyra scanner, according to a freely available protocol (Alfaro-Almagro et al., 2018). All imaging parameters have been reported previously (Cox, Lyall, et al., 2019; Cox, Ritchie, et al., 2019). T1-weighted MPRAGE and T2-weighted FLAIR volumes were acquired in sagittal orientation at 1 × 1 × 1 mm and 1.05 × 1 × 1 mm resolution, respectively. The dMRI acquisition comprised a spin-echo echo-planar sequence with 10 T2-weighted ($b \approx 0$ s mm^{-2}) baseline volumes, 50 $b = 1000$ s mm^{-2} and 50 $b = 2000$ s mm^{-2} diffusion weighted volumes, with 100 distinct diffusion-encoding directions and 2 mm isotropic voxels.

The IDPs in this study included total brain volume, grey matter volume, subcortical volumes (accumbens, amygdala, caudate, hippocampus, pallidum, putamen, thalamus), and tract-averaged fractional anisotropy (FA), mean diffusivity (MD), intra-cellular volume fraction (ICVF), isotropic or free water volume fraction (ISOVF), orientation dispersion index (OD), diffusion principal axis strengths (L1-3), and diffusion tensor mode (MO) of the following white matter tracts: acoustic radiation, anterior thalamic, cingulum gyrus, and parahippocampal, corticospinal, forceps major and minor, inferior fronto-occipital, inferior longitudinal, middle cerebellar peduncle, medial lemniscus, posterior thalamic, superior longitudinal, superior thalamic, and uncinate.

Analysis

Workflow

Heart Disease Filtering

To filter out patients with severe cardiovascular pathology, we identified all patients with any ischaemic heart disease, non-ischaemic cardiomyopathy, valvular disease, or significant

arrhythmia. We derived these diagnoses through a combination of self-reported answers at baseline interview, UK Biobank algorithmically-computed outcomes, and linked HES data codes, reported in detail previously (Raisi-Estabragh, Jaggi, et al., 2021). 1311 of the 13709 subjects that passed initial filtering for completeness had at least one of the above conditions.

Brain Disease Filtering

To align with previous studies and observe only non-pathological variation in cognitive function and brain structure (Cox, Lyall, et al., 2019), we removed all patients with the following diseases provided via self-report during the baseline UK Biobank session: dementia, parkinsons, other chronic/ neurodegenerative, Guillain-Barré, multiple sclerosis, other demyelinating, stroke, brain haemorrhage, brain/intracranial abscess, cerebral aneurysm, cerebral palsy, encephalitis, epilepsy, head injury, infection of nervous system, ischaemic stroke, meningioma (benign), meningitis, motor neuron disease, neurological injury/trauma, spina bifida, subdural haematoma, subarachnoid haemorrhage, transient ischaemic attack, brain cancer, and meningeal cancer. 524 of the 13709 subjects that passed initial filtering for completeness had at least one of the above conditions.

Dimensionality Reduction

CMR Radiomics PCA

To select the number of robust principal components to retain for downstream analysis, we ran 10-fold cross validation over the dataset, computed principal components on the training data, computed the explained variance in the held-out data, plotted the explained variance, and found the elbow in this curve, the point after which the explained variance stops decreasing across PCs.

Here we offer interpretations of the loadings of the principal components to understand their significance (**Supplementary Table 4**). The first PC has large weights for myocardial volume, surface area, and several global texture features correlated with myocardial size. The second PC has large weights for measures of the centre of the myocardial voxel intensity distribution (e.g. mean, median) with end-systolic intensities having higher weights relative to end-diastolic. The third PC has large negative weights for measures of the variability of the myocardial textures

(e.g. entropy, contrast). Such that the value of the third PC correlates with complexity, we used the negative of its PC scores.

Brain MRI IDP PCA Interpretation

For the Factor Analysis, tract measures (left and right) were entered separately into this analysis, correlated residuals between the left and right of each tract and between some other tracts were allowed.

We offer interpretations of the loadings of the first three unrotated principal components of the Brain MRI IDPs (**Supplementary Table 6**). The first PC has high weightings for ICVF and FA of the fasciculi and thalamic tracts. In previous literature, low FA and high MD have been associated with greater age, elevated vascular risk, and lower processing speed (Cox et al., 2016; Cox, Lyall, et al., 2019; Penke et al., 2010). The second PC has large negative weightings for FA of the corticospinal tracts. We took the negative of the second PC scores such that the second PC increases with greater FA. The third has high weightings for volumes, suggesting a factor capturing size.

Heart-Brain Joint Factor Interpretation

To choose the number of CCA modes to keep, we performed 10-fold cross validation on the subjects. For each training fold, we computed ten CCA modes and extracted their loadings. We then applied the loadings to the held-out testing data and found the correlation of the heart and brain components in the held-out data. We then plotted the correlation and chose the number of modes to the left of the elbow, or before the correlation in the modes levels off. Like the PCA method, this cross-validation method ensures that we choose a number of features with reasonable explained variance in unseen UK Biobank subjects.

To assess what features are most important to each mode, we found the correlation of the component scores with the features of each dataset (**Supplementary Table 7**). We found that most interesting associations were negative for the first two modes, so we inverted the sign of their component scores for clarity. The scores of the first mode correlate with most volume measures from both the heart and brain imaging. The scores of the second mode correlate with myocardial intensity in end-systole and correlate with some measures of volume of the brain

image (notably grey matter volume) and many measures of white matter integrity (ICVF and FA) in the thalamic tracts. This mode also has a strong negative correlation with white matter hyperintensity volume. We expect both volumes to decrease and FA to decrease in neurovascular pathology, so this mode likely correlates with neurovascular health (Cox, Lyall, et al., 2019). The scores of the third mode correlate with myocardial intensity in end-diastole, and they also correlate with MD.

Imaging Deconfounding

Both of the imaging datasets generate features sensitive to the size of patients and their position in the scanner. To remove potential confounding on downstream analyses, we regressed all latent variables for the imaging datasets on the imaging confounders and performed future analyses on the residuals of this regression. For the cardiac imaging latents, we regressed each latent onto z-scored body surface area (BSA). We regressed each brain imaging latent onto z-scored head size and the head position in the scanner (X, Y, and Z coordinates). We regressed the components of the joint factors on both the heart and brain imaging confounders because the joint analysis can induce correlations with imaging conditions from either dataset. In analyses that use individual imaging features, we perform this deconfounding on individual features rather than the latent features. Since the uncorrected values may be of interest in some contexts, we report both the uncorrected and corrected values for all analyses in the **Supplementary Tables**.

Linear Models

In **Figure 2, Supplementary Tables 10, 11**, we report the results of linearly modelling each latent measure as a function of each other latent measure controlling for sex and age. We report additional R^2 as the amount of variance that the independent latent measure explains in the dependent latent measure above what is already explained by the covariates.

Mediation Modelling

Since mediation modelling has been described in detail elsewhere (MacKinnon et al., 2007), we will provide a brief explanation of the procedure in the context of this study. We first regress a potential mediator, say grey matter, on gVRF. Label this coefficient ***a***. We then regress g on both

the mediator and gVRF. The coefficient for the mediator is ***b***, and the coefficient for gVRF is ***c'***. The indirect effect of gVRF on g via the mediator is ***a*b***, and the direct effect of VRF on g is ***c'***. The magnitude of the indirect effect indicates the degree to which a mediator explains the observed association between gVRF and g. The total effect is $c=c'+a*b$. The percent mediation is the ratio of the indirect effect and the total effect: $a*b/(c'+a*b)$.

Along with single mediation, we also performed and report parallel and sequential multiple mediation models. We considered two mediators in each multiple mediation model. The first mediator was always Heart PC2 (mediator 1), and we used the brain latents as the second mediator (mediator 2). In parallel multiple mediation, the mediator 1 is not explicitly considered as a predictor for mediator 2 while it is considered in sequential mediation. Therefore, $c=c'+a_1*b_1+a_2*b_2$ in parallel mediation, and $c=c'+a_1*b_1+a_2*b_2+a_1*m*b_2$ in sequential mediation, where ***m*** is the coefficient of the mediator 2 regressed on mediator 1. Comparing the indirect effect from the parallel multiple mediation to the single mediation allows for one to assess how unique the g associations for mediator 1 and mediator 2 are. Similarly, comparing the parallel multiple mediation and the sequential multiple mediation allows one to assess the uniqueness of the gVRF association for mediator 2. In other words, parallel multiple mediation measures how much mediator 1 mediates the association between gVRF and mediator 2.

Packages

For gVRF, g, gMD, and gFA extraction, we performed confirmatory factor analysis (CFA) using 'cfa' from the lavaan R package (Rosseel, 2012). For PCA of the CMR radiomics and brain IDPs, we used prcomp of the base R stats package. We performed CCA using 'CCA' from the scikit-learn cross-decomposition module in Python (Pedregosa et al., 2011). We performed all linear modelling with lavaan in R and report bootstrapped estimates of confidence intervals, method previously reported (Rosseel, 2012). We control for multiple hypothesis testing across all linear models by performing a Benjamini-Hochberg False Discovery Rate (BH-FDR) adjustment for all latent variable p-values (Benjamini & Hochberg, 1995). We performed propensity score matching using 'matchit' (Ho et al., 2011). We used a logistic regression distance metric and a calliper of 0.05. We matched men and women separately and then recombined. For the mediation models, we performed fitting and bootstrapping via lavaan in R and report the direct

effect, indirect effects, total effect, and percent mediation (Rosseel, 2012). In all models, we adjusted for sex and age, report both raw and deconfounded results, and correct for multiple hypothesis testing via a BH-FDR correction.

References

Alfaro-Almagro, F., Jenkinson, M., Bangerter, N. K., Andersson, J. L. R., Griffanti, L., Douaud, G., Sotiropoulos, S. N., Jbabdi, S., Hernandez-Fernandez, M., Vallee, E., Vidaurre, D., Webster, M., McCarthy, P., Rorden, C., Daducci, A., Alexander, D. C., Zhang, H., Dragonu, I., Matthews, P. M., … Smith, S. M. (2018). Image processing and Quality Control for the first 10,000 brain imaging datasets from UK Biobank. *NeuroImage*, *166*, 400–424.

Attar, R., Pereañez, M., Gooya, A., Albà, X., Zhang, L., de Vila, M. H., Lee, A. M., Aung, N., Lukaschuk, E., Sanghvi, M. M., Fung, K., Paiva, J. M., Piechnik, S. K., Neubauer, S., Petersen, S. E., & Frangi, A. F. (2019). Quantitative CMR population imaging on 20,000 subjects of the UK Biobank imaging study: LV/RV quantification pipeline and its evaluation. *Medical Image Analysis*, *56*, 26–42.

Bai, W., Sinclair, M., Tarroni, G., Oktay, O., Rajchl, M., Vaillant, G., Lee, A. M., Aung, N., Lukaschuk, E., Sanghvi, M. M., Zemrak, F., Fung, K., Paiva, J. M., Carapella, V., Kim, Y. J., Suzuki, H., Kainz, B., Matthews, P. M., Petersen, S. E., … Rueckert, D. (2018). Automated cardiovascular magnetic resonance image analysis with fully convolutional networks. *Journal of Cardiovascular Magnetic Resonance: Official Journal of the Society for Cardiovascular Magnetic Resonance*, *20*(1), 65.

Bai, W., Suzuki, H., Huang, J., Francis, C., Wang, S., Tarroni, G., Guitton, F., Aung, N., Fung, K., Petersen, S. E., Piechnik, S. K., Neubauer, S., Evangelou, E., Dehghan, A., O’Regan, D. P., Wilkins, M. R., Guo, Y., Matthews, P. M., & Rueckert, D. (2020). A population-based phenome-wide association study of cardiac and aortic structure and function. *Nature Medicine*, *26*(10), 1654–1662.

Benjamini, Y., & Hochberg, Y. (1995). Controlling the false discovery rate: A practical and powerful approach to multiple testing. *Journal of the Royal Statistical Society*, *57*(1), 289–300.

Carré, A., Klausner, G., Edjlali, M., Lerousseau, M., Briend-Diop, J., Sun, R., Ammari, S., Reuzé, S., Alvarez Andres, E., Estienne, T., Niyoteka, S., Battistella, E., Vakalopoulou, M., Dhermain, F., Paragios, N., Deutsch, E., Oppenheim, C., Pallud, J., & Robert, C. (2020). Standardization of brain MR images across machines and protocols: bridging the gap for MRI-based radiomics. *Scientific Reports*, *10*(1), 12340.

Cox, S. R., Lyall, D. M., Ritchie, S. J., Bastin, M. E., Harris, M. A., Buchanan, C. R., Fawns-Ritchie, C., Barbu, M. C., de Nooij, L., Reus, L. M., Alloza, C., Shen, X., Neilson, E., Alderson, H. L., Hunter, S., Liewald, D. C., Whalley, H. C., McIntosh, A. M., Lawrie, S. M., … Deary, I. J. (2019). Associations between vascular risk factors and brain MRI indices in UK Biobank. *European Heart Journal*, *40*(28), 2290–2300.

Cox, S. R., Ritchie, S. J., Fawns-Ritchie, C., Tucker-Drob, E. M., & Deary, I. J. (2019). Structural brain imaging correlates of general intelligence in UK Biobank. *Intelligence*, *76*, 101376.

Cox, S. R., Ritchie, S. J., Tucker-Drob, E. M., Liewald, D. C., Hagenaars, S. P., Davies, G., Wardlaw, J. M., Gale, C. R., Bastin, M. E., & Deary, I. J. (2016). Ageing and brain white matter structure in 3,513 UK Biobank participants. *Nature Communications*, *7*, 13629.

Ho, D., Imai, K., King, G., & Stuart, E. A. (2011). MatchIt: Nonparametric Preprocessing for Parametric Causal Inference. *Journal of Statistical Software*, *42*, 1–28.

MacKinnon, D. P., Fairchild, A. J., & Fritz, M. S. (2007). Mediation analysis. *Annual Review of Psychology*, *58*, 593–614.

Pedregosa, F., Varoquaux, G., Gramfort, A., Michel, V., Thirion, B., Grisel, O., Blondel, M., Prettenhofer, P., Weiss, R., Dubourg, V., & Others. (2011). Scikit-learn: Machine learning in Python. *The Journal of Machine Learning Research*, *12*, 2825–2830.

Penke, L., Muñoz Maniega, S., Murray, C., Gow, A. J., Hernández, M. C. V., Clayden, J. D., Starr, J. M., Wardlaw, J. M., Bastin, M. E., & Deary, I. J. (2010). A general factor of brain white matter integrity predicts information processing speed in healthy older people. *The Journal of Neuroscience: The Official Journal of the Society for Neuroscience*, *30*(22), 7569–7574.

Petersen, S. E., Aung, N., Sanghvi, M. M., Zemrak, F., Fung, K., Paiva, J. M., Francis, J. M., Khanji, M. Y., Lukaschuk, E., Lee, A. M., Carapella, V., Kim, Y. J., Leeson, P., Piechnik, S. K., & Neubauer, S. (2017). Reference ranges for cardiac structure and function using cardiovascular magnetic resonance (CMR) in Caucasians from the UK Biobank population cohort. *Journal of Cardiovascular Magnetic Resonance: Official Journal of the Society for Cardiovascular Magnetic Resonance*, *19*(1), 18.

Petersen, S. E., Matthews, P. M., Francis, J. M., Robson, M. D., Zemrak, F., Boubertakh, R., Young, A. A., Hudson, S., Weale, P., Garratt, S., Collins, R., Piechnik, S., & Neubauer, S. (2016). UK Biobank's cardiovascular magnetic resonance protocol. *Journal of Cardiovascular Magnetic Resonance: Official Journal of the Society for Cardiovascular Magnetic Resonance*, *18*(1), 1–7.

Raisi-Estabragh, Z., Harvey, N. C., Neubauer, S., & Petersen, S. E. (2021). Cardiovascular magnetic resonance imaging in the UK Biobank: a major international health research resource. *European Heart Journal Cardiovascular Imaging*, *22*(3), 251–258.

Raisi-Estabragh, Z., Jaggi, A., Gkontra, P., Mccracken, C., Aung, N., Munroe, P. B., Neubauer, S., Harvey, N. C., Lekadir, K., & Petersen, S. E. (2021). Cardiac magnetic resonance radiomics reveal differential impact of sex, age, and vascular risk factors on cardiac structure and myocardial tissue. *Frontiers in Cardiovascular Medicine*, *8*, 1972.

Rosseel, Y. (2012). lavaan: An R Package for Structural Equation Modeling. *Journal of Statistical Software*, *48*, 1–36.

van Timmeren, J. E., Cester, D., Tanadini-Lang, S., Alkadhi, H., & Baessler, B. (2020). Radiomics in medical imaging-"how-to" guide and critical reflection. *Insights into Imaging*, *11*(1), 91.

Supplementary Figures

Supplementary Figure 1

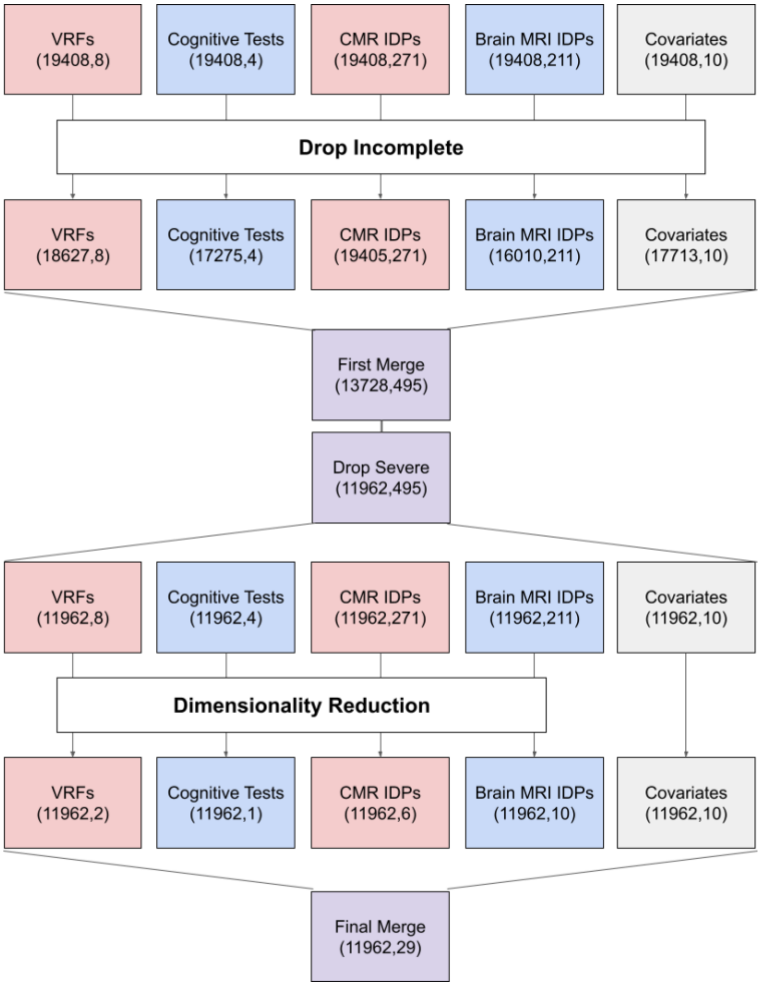

Supplementary Figure 1: Data Preparation Workflow

The flow of subjects and variables through data preparation. Each box represents the number of subjects and the number of variables after the most recent action was taken. We began with 19408 subjects with complete CMR imaging and pulled their data for the other categories. We then dropped incomplete subjects for each category and removed all subjects with a severe cardiovascular or brain disease diagnosis. We then separately conducted factor analysis (and joint factor analysis not illustrated here for clarity) and finally merged all latent variables and covariates for downstream modelling. Characteristics of final cohort in **Supplementary Table 1**.

Supplementary Figure 2

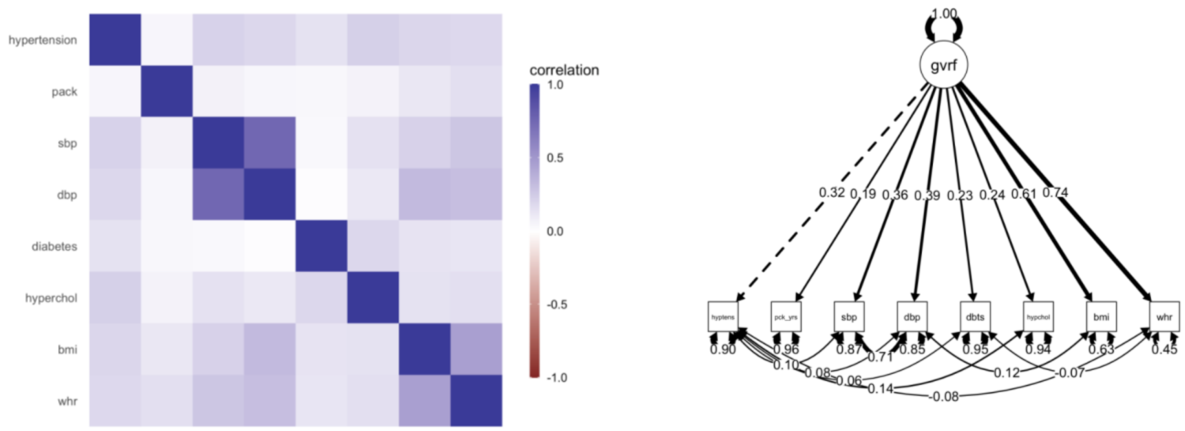

Supplementary Figure 2: CFA for Vascular Risk

A correlation heatmap and factor loadings for all vascular risk variables. Fit measures reported in **Supplementary Table 2**.

Supplementary Figure 3

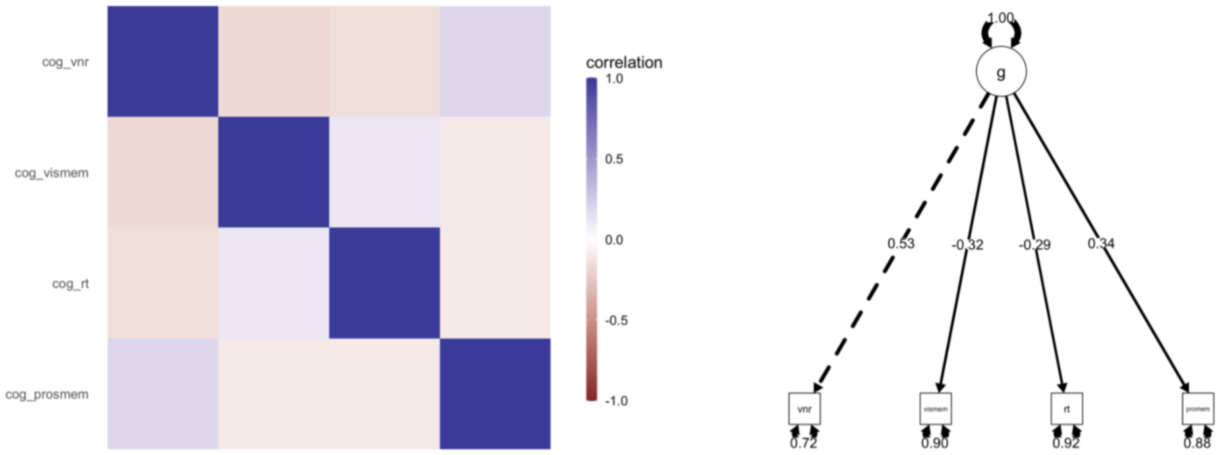

Supplementary Figure 3: CFA for Cognitive Function

A correlation heatmap and factor loadings for the cognitive exams. Fit measures reported in **Supplementary Table 3**.

Supplementary Figure 4

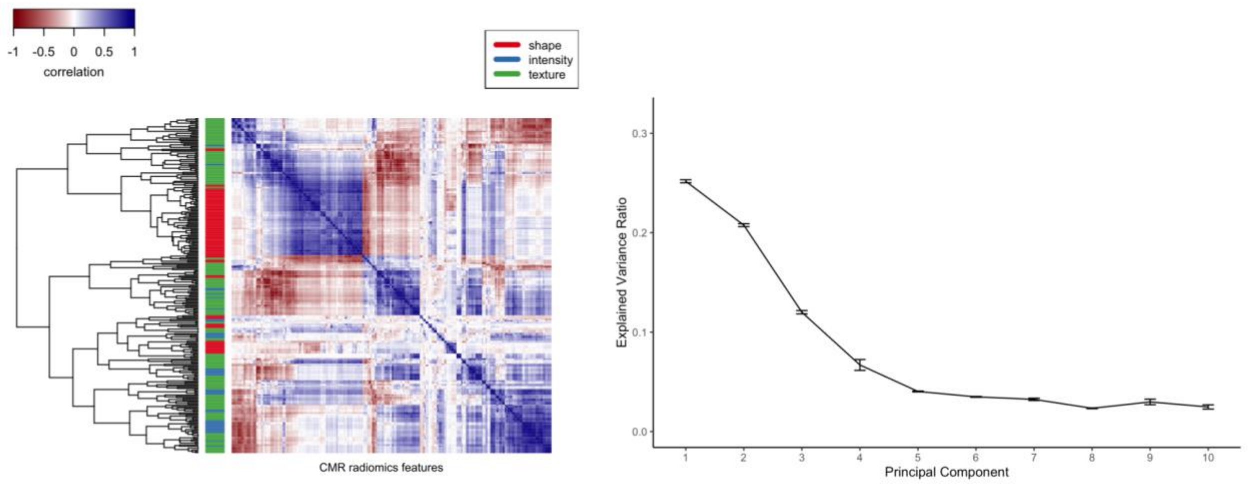

Supplementary Figure 4: PCA for CMR Radiomics

A correlation heatmap of CMR Radiomics features (sorted by a complete linkage dendrogram) with a colorbar for the original feature type. Percent variance explained of the top ten principal components of the features, error bars represent standard error over 10-fold cross validation. Individual feature loadings for each component are reported in **Supplementary Table 4**.

Supplementary Figure 5

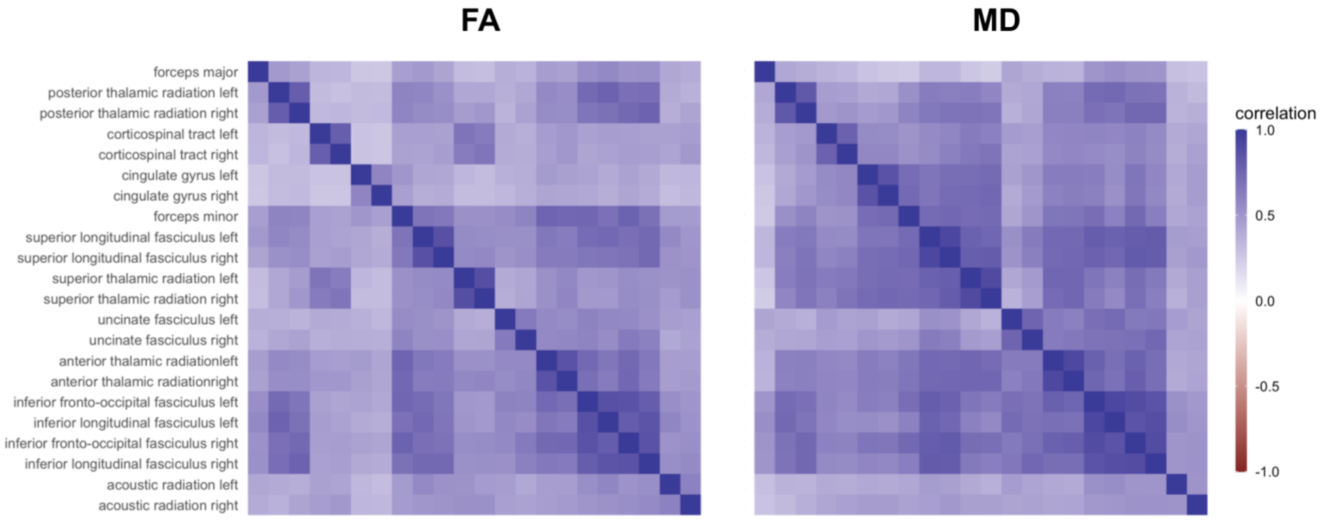

Supplementary Figure 5: CFA for FA and MD

A correlation heatmap of FA and MD IDPs. Fit measures and variable loadings are reported in **Supplementary Table 5**.

Supplementary Figure 6

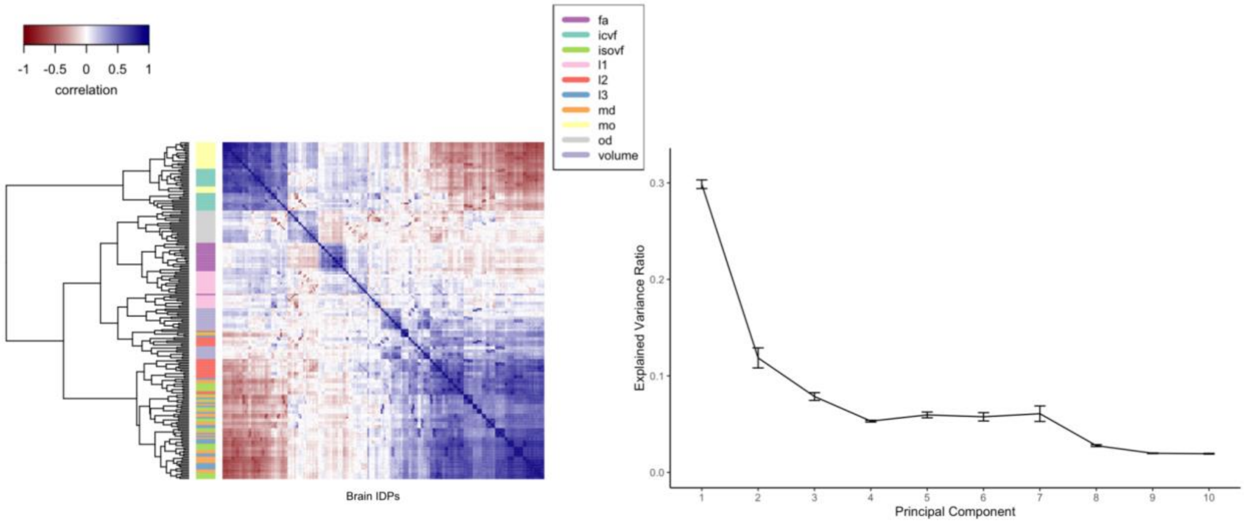

Supplementary Figure 6: PCA for Brain IDPs

A correlation heatmap of brain MR IDPs (sorted by a complete linkage dendrogram) with a colorbar for the original feature type. Percent variance explained of the top ten principal components of the features, error bars represent standard error over 10-fold cross validation. Individual feature loadings for each component are reported in **Supplementary Table 6**.

Supplementary Figure 7

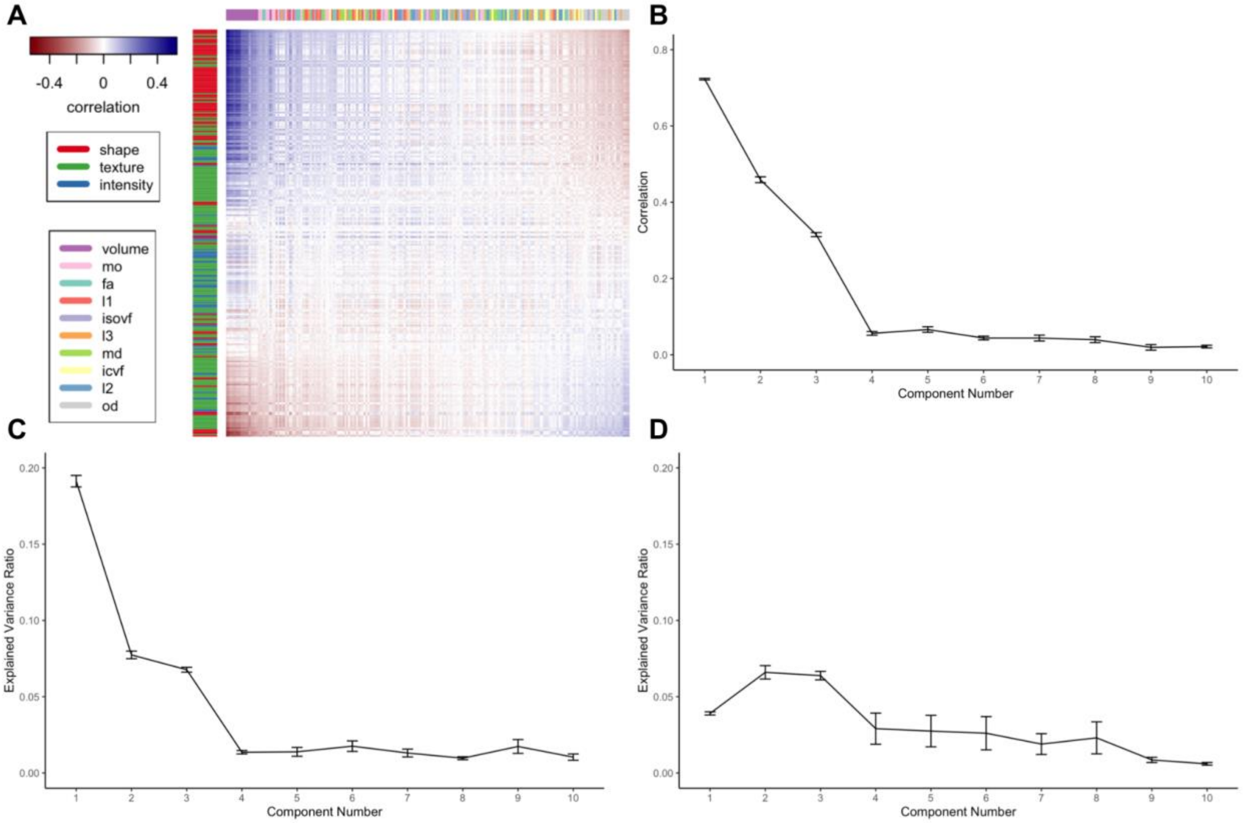

Supplementary Figure 7: CCA for Heart and Brain IDPs

(A) A correlation heatmap of the CMR Radiomics (rows) and Brain IDPs (columns). The heatmap columns and rows have been ordered by the weight of each feature in the loadings of the first CCA mode. (B) The correlation between the first ten CCA modes for the heart and brain imaging data in held-out data. The errors represent standard error over 10-fold cross validation. (C) The variance explained in held-out data by the heart component of each CCA mode. (D) The variance explained in held-out data by the brain component of each CCA mode. Individual feature loadings are reported in **Supplementary Table 7**.

Supplementary Figure 8

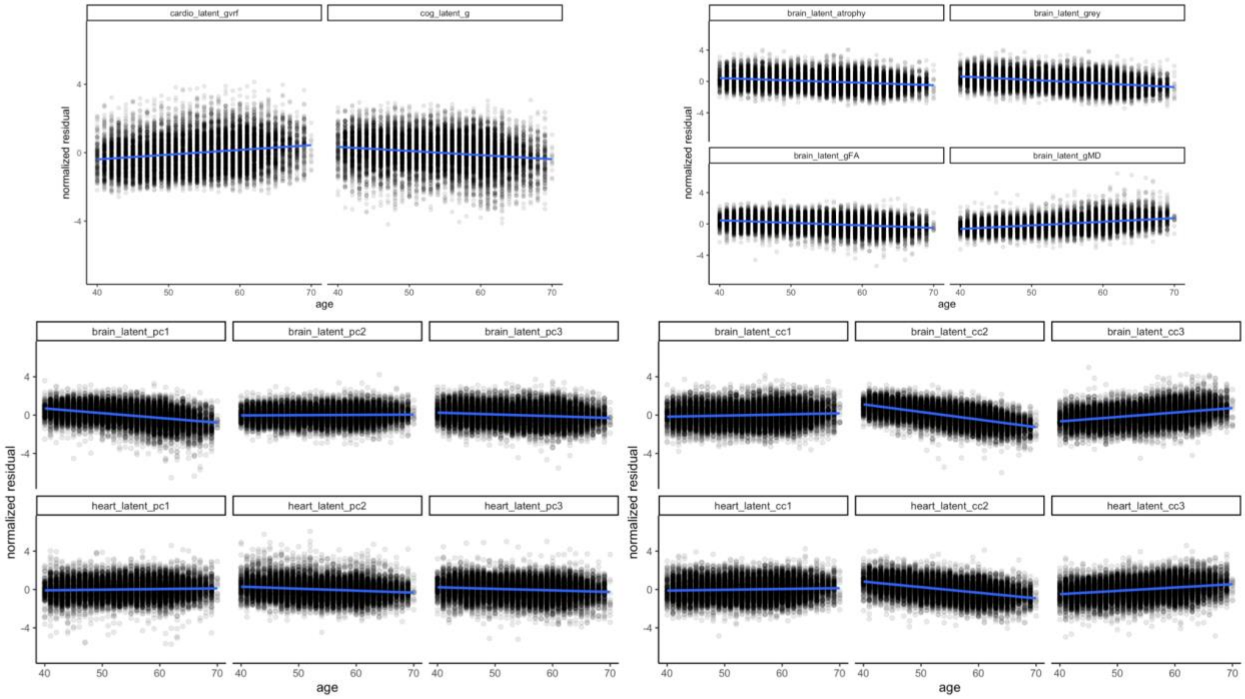

Supplementary Figure 8: Age Associations

Plot of each latent measure against age. Each latent has been deconfounded from imaging parameters as described in **Supplementary Methods**. Linear model estimates reported in **Supplementary Tables 8 and 9**.

Supplementary Figure 9

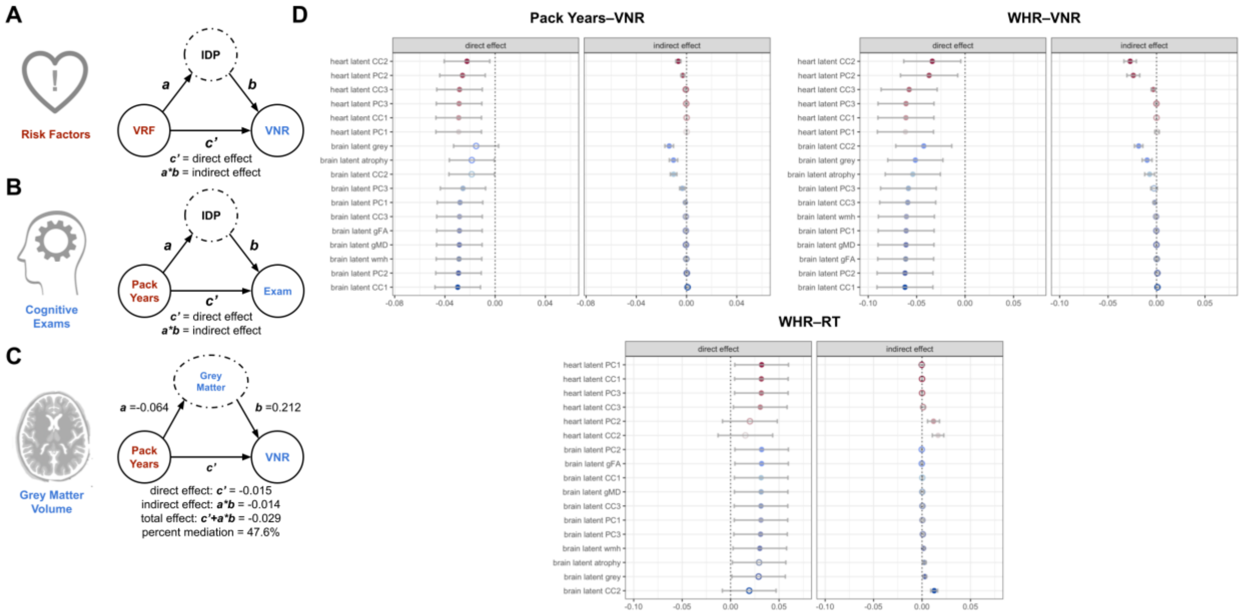

Supplementary Figure 9: Latent Single Mediation Modelling of VRF-Cognitive Pairs

We performed serial mediation modelling for all latent imaging measures for each VRF-cognitive exam pair. (A) Schematic for a mediation model using different VRFs as the independent variable rather than gVRF. We tested all VRFs. (B) Schematic for a mediation model using cognitive exams as the dependent variable rather than g. We tested all exams. (C) Example mediation model for an individual latent factor and an example pair of VRF and cognitive exam. Confidence intervals for all coefficient estimates in **Supplementary Table 21**. (D) Direct and indirect effects for three significant VRF-exam pairs. Latents ordered by indirect effect size and separated by organ. RT shows lower values for better performance while VNR shows higher values for higher performance.

Supplementary Figure 10

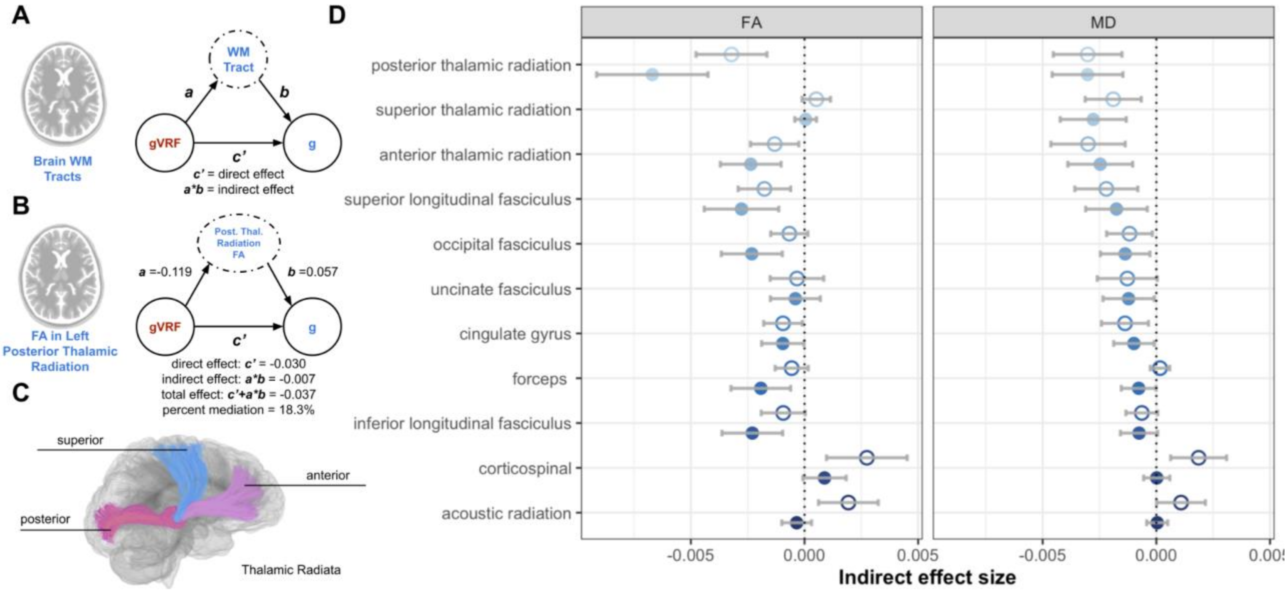

Supplementary Figure 10: White Matter Tract Mediation Modelling

We focus on serial mediation modelling for individual white matter tract quantifiers. (A) Schematic for the modelling procedure for the brain white matter tract quantifiers. (B) Example mediation model for an individual feature, fractional anisotropy of the posterior thalamic radiation. Confidence intervals reported in **Supplementary Table 25**. (C) Illustration of the key white matter tracts with large indirect effects for both FA and MD. (D) Indirect effects for all tested FA and MD measures.

Supplementary Table Captions

Supplementary Table 1: Summary statistics of the final cohort

Supplementary Table 2: Loadings and Fit Metrics for CFA of Vascular Risk Factors (cfi: comparative fit index, tli: Tucker-Lewis index, rmsea: root mean square error of approximation, srmr: standardised root mean square residual)

Supplementary Table 3: Loadings and Fit Metrics for CFA of Cognitive Exams

Supplementary Table 4: PCA Loadings for CMR Radiomics

Supplementary Table 5: Loadings and Fit Metrics for CFA of Fractional Anisotropy and Mean Diffusivity

Supplementary Table 6: PCA Loadings for all Brain MRI IDPs

Supplementary Table 7: CCA Loadings for CMR Radiomics and Brain MRI IDPs

Supplementary Table 8: Association of each Latent Factor with Age and Sex, Raw (ci.lower: lower 95% confidence interval, ci.upper: upper 95% confidence interval, pvalue_adj: BH-FDR adjusted p-value)

Supplementary Table 9: Association of each Latent Factor with Age and Sex, Deconfounded

Supplementary Table 10: Coefficients and Metrics for Pairwise Linear Modelling of all Latents, Raw (total.r: R-squared including covariates, additional.r: additional R-squared above covariates)

Supplementary Table 11: Coefficients and Metrics for Pairwise Linear Modelling of all Latents, Deconfounded

Supplementary Table 12: Propensity Score Matching un-paired t-tests (control mean: mean from group with zero VRFs, at risk mean: mean from group with 4+ VRFs, count of instances reported instead of mean for binary variables, diff: difference of means, percent.diff: average percent difference of means with control as denominator, significant: whether adjusted pvalue below 0.05)

Supplementary Table 13: Single Mediation Model Coefficients and Metrics for all Latents, Raw (lhs: left hand side, op: operation, rhs: right hand side, label: mediation model variable label, one of a, b, c, de: direct effect, ie: indirect effect, te: total effect, pm: percent mediation, est.std: standardised estimate)

Supplementary Table 14: Single Mediation Model Coefficients and Metrics for all Latents, Deconfounded

Supplementary Table 15: Association of BMI with Heart PC2 controlling for heart size, mediation of the BMI-VNR association by Heart PC2, and mediation of the gVRF-g association by Heart PC2 covarying for BMI

Supplementary Table 16: Multiple Parallel Mediation Model Coefficients and Metrics for all Latents, Raw (ie1: indirect effect of mediator 1, ie2: indirect effect of mediator 2)

Supplementary Table 17: Multiple Parallel Mediation Model Coefficients and Metrics for all Latents, Deconfounded

Supplementary Table 18: Multiple Sequential Mediation Model Coefficients and Metrics for all Latents, Raw (ie3: indirect effect through mediators 1 and 2 in sequence, see **Supplementary Methods** for more details)

Supplementary Table 19: Multiple Sequential Mediation Model Coefficients and Metrics for all Latents, Deconfounded

Supplementary Table 20: Single Mediation Model Coefficients and Metrics for all Latents and all significant VRF-cognitive pairs, Raw

Supplementary Table 21: Single Mediation Model Coefficients and Metrics for all Latents and all significant VRF-cognitive pairs, Deconfounded

Supplementary Table 22: Single Mediation Model Coefficients and Metrics for all individual CMR radiomics features, Raw (type: category of CMR radiomics feature)

Supplementary Table 23: Single Mediation Model Coefficients and Metrics for all individual CMR radiomics features, Deconfounded

Supplementary Table 24: Single Mediation Model Coefficients and Metrics for all individual Brain MRI IDPs, Raw (type: category of brain MRI IDP)

Supplementary Table 25: Single Mediation Model Coefficients and Metrics for all individual Brain MRI IDPs, Deconfounded

Supplementary Table 26: Single Mediation Model Coefficients and Metrics for all Bai, et al. CMR features, Deconfounded
